# Effects of Crystallinity and Branched Chain on Thermal Degradation of Polyethylene: A SCC-DFTB Molecular Dynamics Study

**DOI:** 10.3390/polym16213038

**Published:** 2024-10-29

**Authors:** Shumao Zeng, Diannan Lu, Rui Yang

**Affiliations:** Department of Chemical Engineering, Tsinghua University, Beijing 100190, China; zsm22@mails.tsinghua.edu.cn

**Keywords:** polyethylene thermal degradation, crystallinity, branched chain, molecular dynamics simulations, density functional theory calculation

## Abstract

As a widely used plastic, the aging and degradation of polyethylene (PE) are inevitable problems, whether the goal is to prolong the life of PE products or address the issue of white pollution. Molecular simulation is a vital scientific tool in elucidating the mechanisms and processes of chemical reactions. To obtain the distribution and evolution process of PE’s thermal oxidation products, this work employs the self-consistent charge–density functional tight binding (SCC-DFTB) method to perform molecular simulations of the thermal oxidation of PE with different crystallinity and branched structures. We discovered that crystallinity does not affect the thermal oxidation mechanism of PE, but higher crystallinity makes PE more susceptible to cross-linking and carbon chain growth, reducing the degree of PE carbon chain breakage. The branched structure of PE results in differences in free volumes between the carbon chains, with larger pores leading to a concentrated distribution of O_2_ and chemical defects subsequently formed. The breakdown of PE is slowed down when chemical defects are localized in low-density regions of the carbon chain. The specifics and mechanism of PE’s thermal oxidation are clearly revealed in this paper, which is essential for understanding the process in depth and for the development of anti-aging PE products.

## 1. Introduction

As a form of thermoplastic resin that has exceptional corrosion resistance to most acids and alkalis, PE is odorless, chemically stable, non-toxic, and possesses great insulating qualities [1]. Consequently, it finds extensive application in high-frequency insulating materials, containers, wire encapsulation, and packaging materials [2]. Nevertheless, although exhibiting stable chemical characteristics, PE still suffers problems, including thermal oxidation [3,4,5] and photo-oxidation [6,7,8]. The PE chains break due to these oxidation processes, and the chemical defects accelerate the reactions between carbon chains [9,10]. A thorough grasp of the regularity of oxidation will promote the development of superior PE products and help extend its service life.

The thermal oxidation of PE is a common phenomenon during processing and applications. Accordingly, it is crucial to clarify the laws and factors that affect thermal oxidation. PE’s thermal oxidation process has been explored via experiments [11,12,13,14] and simulations [15,16,17,18].

A valuable method for anticipating the results of thermal oxidation is molecular dynamics simulations. Lijuan Liao et al. employed reactive force field (ReaxFF) molecular dynamics to simulate the thermal oxidation of amorphous PE under anaerobic and low O_2_ concentration circumstances [15]. The findings demonstrated that hydroxyl, aldehyde, and carboxyl groups are typical organic products when O_2_ is present. The activation energy of PE thermal oxidation varies from 50 to 450 kJ/mol as the reaction progresses, indicating the complexity of thermal oxidation. However, the effects of crystallinity and branched chain structure on oxidation products were not considered in their work.

Additionally, molecular dynamics simulation is an effective way to investigate the formation mechanism of different products. Lihua Chen et al. [16] investigated the formation pathways and energy barrier of olefins, hydroxyl, aldehydes, and ketones. They revealed that the carboxyl group is dehydrated to generate the ketone carbonyl group, and the dehydration process also produces an O-containing free radical. Due to the O-containing free radical, the carbon chain becomes unstable, leading to chain scission that produces aldehydes and hydroxyl. However, Mingxiao Zhu et al. [17] obtained different results, claiming that when PE is thermally oxidized, the carbon chain breaks first—instead of the H atoms dissociating as Lihua had predicted. Subsequently, O_2_ and the carbon chain breakpoint combine, followed by a dehydration reaction that forms aldehydes. The thermal oxidation mechanism of PE needs further study from a microscopic perspective to resolve this controversy.

The work mentioned above primarily focuses on the thermal oxidation of unbranched PE, although branched chain structures are ubiquitous, resulting in different densities in PE. Jin Woo Park et al. [19] conducted an experimental investigation of the thermal degradation behavior of high-density PE (HDPE), low-density PE (LDPE), and linear low-density PE (LLDPE) from 650 K to 850 K under an inert atmosphere. They discovered that HDPE has the highest stability, whereas LDPE is the most susceptible. They determined that the HDPE, LDPE, and LLDPE degradation activation energies were 333.2–343.2, 187.5–199.1, and 219.2–230.1 kJ/mol, respectively. As the degree of branching grows, so does the reaction order.

Although the above experimental work studied the thermal degradation activation energy of PE with different densities, there is still a lack of research on how density and branched structure affect PE’s thermal oxidation, and the mechanism from a micro perspective remains to be explored. Additionally, the branch length and branch density of HDPE, LDPE, and LLDPE differ, resulting in variations in their crystallinity. The branched structure affects the ease of reaction. Variations in crystallinity cause carbon chains to be arranged differently, which might have an impact on the movement of carbon chain migration [20] and diffusion of O_2_ molecules [21]. Consequently, the thermal oxidation of PE becomes more complicated due to the crystallinity and branched chain structure.

As mentioned above, it is likely that crystallinity and branched chain structure have an important impact on the thermal oxidation behavior of PE. We aim to understand how branching and crystallinity affect the thermal oxidation of PE in a thermodynamic or kinetic way. However, this question is challenging to answer experimentally, and there are still few simulation studies in this area. To close the gaps and provide clarity on the intricate thermal oxidation of PE with various structures, we established four models: high-density crystalline PE (HDPE-C), high-density amorphous unbranched PE (HDPE-A), LDPE with a random and short chain structure, and LLDPE with a regular branched chain structure. By comparing HDPE-C and HDPE-A, the effect of crystallinity can be understood. By comparing HDPE-A, LDPE, and LLDPE, the impact of the branched structure can be understood.

Our research demonstrates that higher crystallinity makes PE more susceptible to cross-linking and carbon chain growth, reducing the degree of PE carbon chain breakage. Although crystallinity and branched structure do not affect the thermal oxidation by affecting the thermodynamic energy barrier of the reaction of PE with O_2_, short and random branched chains contribute to a local pore structure in the carbon chain, leading to the localized distribution of O_2_ and subsequently formed chemical defects, and delaying the chain scission of PE. This will help to inspire the design of more stable polyolefin products, such as controlling the polymerization process to tune the branching structure of PE and processing parameters to tune crystallinity to obtain PE with high stability. This is of great significance for extending the life of polyolefin products. To increase the service life of PE products, it is crucial to design and enhance the molecular structure of PE at the manufacturing level.

## 2. Methods

We utilized the Materials Studio software (version 1.9.4) for model construction, employing molecular dynamics to simulate the thermal oxidation process of PE. Furthermore, the density functional theory (DFT) method was employed to scrutinize the thermodynamic energy landscape of PE reacting with O_2_.

**Figure 1:**
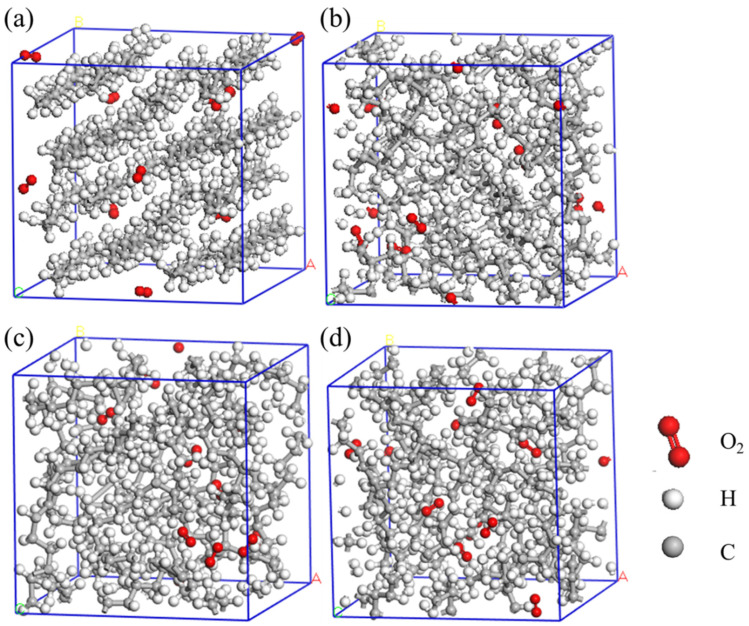
Model construction for (**a**) HDPE-C, (**b**) HDPE-A, (**c**) LDPE, (**d**) LLDPE.

Model construction: In this work, four PE models, i.e., HDPE-C, HDPE-A, LDPE, and LLDPE, were constructed. The comparison of HDPE-C and HDPE-A can reflect the effect of crystallinity on the thermo-oxidative behavior of the PE, whereas the comparison of HDPE-A, LDPE, and LLDPE can show the effect of the branched chains. Three models of HDPE-A, LDPE, and LLDPE were constructed utilizing the Amorphous cell module in Materials Studio. It was configured to have a consistently ordered chain structure for the HDPE-C, which has a density of 0.97 g/cc and is set to 16 carbon chains to prevent interactions between the periodic lattices. When modeling LDPE and LLDPE, the structural details of PE found in Ref. [22] were implemented. LDPE has a branch density of 8CH_3_/200C and a random amount within six branched C atoms, and LLDPE has a branch density of 4CH_3_/200C, with six C atoms in each branch. Both LDPE and LLDPE have densities of 0.92 g/cc. There are no branches in HDPE-A, and the density is 0.94 g/cc. Each of the models has 10 O_2_ molecules and 224 CH_2_ units. Using the PCFF force field describing polymers [23,24,25], structural optimization and thermodynamic relaxation were conducted in the Forcite module. Structural optimization was carried out by employing the conjugate gradient method. The energy convergence standard was 10^−4^ kcal mol^−1^, and the force convergence standard was 0.005 kcal mol^−1^ Å^−1^. After structural optimization, thermodynamic relaxation was performed. To keep the set density constant, only NVT ensemble relaxation was used. The relaxation time was 400 ps, with the temperature 323 K. Berendsen thermostat was adopted, and the attenuation constant was set to 0.1 ps. All the structures are ultimately shown in Figure 1.

Ab initio molecular dynamics (AIMDs) calculation: The free source program CP2K was applied to complete the calculation, and the SCC-DFTB approach was chosen. In classical DFT calculations, the exchange-correlation is not exact, or at least we do not know the exact form yet, and thus, some approximations are needed to obtain the best value possible. To achieve the exchange-correlation contribution, which is difficult to determine, SCC-DFTB fitted the system energy expression defined by electron density, applying the present functional, and a second-order expansion was conducted on the exchange-correlation term. Accordingly, the energy of a multi-electron system can be calculated as [26]:

(1) $$E=\sum_{i}^{occ}\left\langle \Psi_{i}|\hat{H_{0}}|\Psi_{i}\right\rangle +\frac{1}{2}\sum_{\alpha ,\beta }^{N}\gamma_{\alpha \beta }\Delta q_{\alpha }\Delta q_{\beta }+E_{rep}$$

where E is the total energy of the system, and the first sum is over the occupied Kohn–Sham eigenstates $\Psi_{i}$. *H_0_* is the Hamiltonian operator, which generally includes the kinetic energy operator, the external field potential operator, and the electron–electron interaction operator. $E_{rep}$ represents the repulsive two-particle interaction. The second sum is the final second-order energy term. $\Delta q_{\alpha }$ and $\Delta q_{\beta }$ are fluctuations of the *α* and *β* atomic charge. $\gamma_{\alpha \beta }$ is an integral related to the normalized radial dependence of the density fluctuation on atoms *α* and *β*, which can be written as a function of the Hubbard parameter and distance between the two atoms:

(2) $$\gamma_{\alpha \beta }=\gamma_{\alpha \beta }(U_{\alpha ,}U_{\beta },\left|\vec{R_{\alpha }}-\vec{R_{\beta }}\right|)$$

As a result, SCC-DFTB could basically get as close to the accuracy of DFT as possible, while for substantial systems, the computation speed was increased. It is reasonable to believe that SCC-DFTB is also applicable in PE and O_2_ systems, as it has been successfully applied thus far in systems such as metal oxide and water systems [27], light element covalent compound systems [28], organic small molecule systems [29], and C-containing polymers [30]. Canonical sampling through velocity rescaling (CSVR) heat bath was employed, with the time constant set to 80 fs. The NVT ensemble was implemented throughout with a simulation temperature of 2400 K to speed up the thermal oxidation. In molecular simulation, reaction paths with lower energy are more easily sampled by the system. Therefore, low-energy barrier paths usually dominate the reaction mechanism, and high temperature only increases the probability of the system crossing the high-energy barrier. In this case, high temperature only accelerates the sampling efficiency of each reaction rather than changing the reaction mechanism of the system. This shows that in high-temperature simulation, the relative weights of different reaction paths will generally remain similar to those at room temperature; thus, the high-temperature simulation is feasible. The simulation duration was 200 ps with a time step of 0.25 fs. Numerous earlier studies have demonstrated that raising the temperature in a polymer degradation or oxidation scenario can accelerate the reaction without changing the process’s mechanism [31,32,33,34,35]. Simultaneously, we considered spin multiplicity in the computation by setting the RELAX_MULTIPLICITY parameter to 0.01. Long-range van der Waals interactions were also included in the SCC-DFTB technique. The Python application was executed with Anaconda software (version 2.6.2) to process the atomic coordinate data obtained from the molecular simulation. When assessing the bonding status between two atoms, the criteria involved the van der Waals radius, with a bond presumed to have occurred if the distance between two atoms was less than 0.6 times the sum of their van der Waals radii. Determining whether bonds are formed between atoms occurs by counting the amount of various fragments in the system. In statistics, the following expression is used to calculate the amount of fragments [31]:

(3) $$N_{total}=\sum_{1}^{n}N_{i}$$

$N_{total}$ is the total amount of n fragments at any moment, $N_{i}$ is the specific amount of species, *i*.

In order to study the diffusion behavior of O_2_ in amorphous PE (the oxygen diffusion behavior of crystalline PE has been reported in Ref. [36]) and the interaction energy between PE and O_2_, we performed classical molecular dynamics simulations using the PCFF force field in the Forcite module of Materials Studio software. After the NVT relaxation, in order to obtain the mean square displacement (MSD) and the interaction energy between PE and O_2_, we conducted dynamics simulations at 300 K under the NVT ensemble of 4.5 ns and 600 ps, respectively. The following formula is used to calculate the interaction energy [36]:

(4) $$E_{\mathrm{inter}}=(E_{\mathrm{complex}}-E_{O_{2}}-E_{\mathrm{PE}})/N$$

where *E_inter_* is the interaction energy, *E_complex_* is the energy of the system after PE and O_2_ are mixed, $E_{O_{2}}$ is the energy of all O_2_ molecules, *E_PE_* is the energy of PE, *N* is the number of O_2_ molecules, and each energy is the average total energy of the last 100 ps obtained by relaxing each system separately.

Quantum chemical (QC) computations: The DFT method was chosen to execute subsequent computations of the Gibbs free energy change (ΔG) in Gaussian09 software. Geometry optimization and frequencies are carried out by the B3LYP functional with a 6-311g+(d,p) basis set. The convergence criteria are the default criteria of the Gaussian software. There was no imaginary frequency for all the reactants and products, proving that all the structures were stable. Single point energies (SPEs) for all the structures were calculated at the M06-2x/aug-cc-pVDZ level. In this work, unbranched and branched PE with C atoms between 1 and 6 were simulated. To make the non-periodic model closer to real PE, the number of C atoms in the main chain was nine in all models.

Free volume calculation: In order to reveal the size and shape of the free volume in PE more clearly, we calculated the free volume of three amorphous PEs using Multiwfn software (version: 3.8) [37]. The calculations were performed based on the promolecular electron density, and the criterion for the division of grids was set to an electron density of less than 0.001 a.u., with a spacing of 0.2 Bohr, and the extension distance was set to 0.

## 3. Results and Discussions

This article first simulates the thermal oxidative degradation of PE, giving the main products and their distribution, and then discusses the reaction mechanism. After analyzing the distribution of chemical defects and carbon chain density, the effects of crystallinity and branched chain structure on the thermal oxidation behavior of PE are discussed.

### 3.1. Distribution of Oxidation Products of Different PEs

This section compares the thermal oxidation products of the four PEs from the perspective of carbon numbers and types of small molecules and demonstrates the degradation process of PE by showing the change in the amount of C–C bonds.

**Figure 2:**
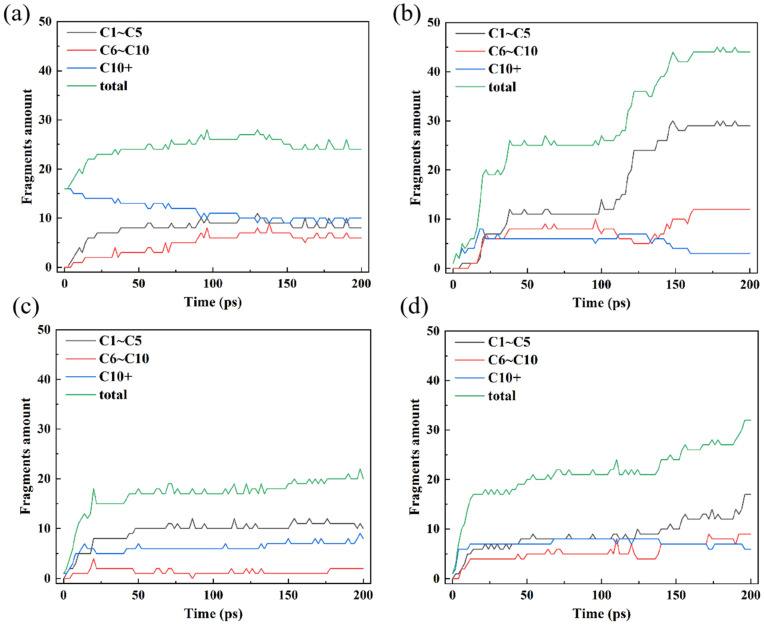
Carbon fragments distribution of (**a**) HDPE-C, (**b**) HDPE-A, (**c**) LDPE, (**d**) LLDPE.

Figure 2 illustrates how C-containing molecular fragments changed over time. For the sake of simplicity and aesthetics, we added up the number of all fragments from C1 to C5 and C6 to C10 before displaying them, which represent small fragments and medium fragments, respectively. The distribution of the quantities of fragment products from C1 to C10 is shown in Figure S1. The results of HDPE-C and HDPE-A demonstrate that crystalline PE is less prone to fragmentation due to bond breakdown. Comparatively, more fragments are formed in HDPE-A, and HDPE-A produces more small fragments. After around 150 ps, the total quantity of fragments in HDPE-C decreases, suggesting that the system is experiencing carbon chain growth reactions. This is due to the restricted mobility of the carbon chain in the HDPE-C system, causing the alkyl radicals to only migrate along the carbon chain and the relatively sluggish movement of the chemical defects.

When HDPE-A, LDPE, and LLDPE are compared, it becomes clear that HDPE-A and LLDPE are more susceptible to oxidation and structural degradation. In both systems, more small fragments are formed. Compared to HDPE-A, LDPE has not undergone as much deterioration. This result is consistent with that in Ref. [12], in which the unbranched M6 sample exhibited a higher carbonyl index than the branched M12 sample during thermal oxidation. A higher carbonyl index means more defects and a greater probability of carbon chain breakage [12].

**Figure 3:**
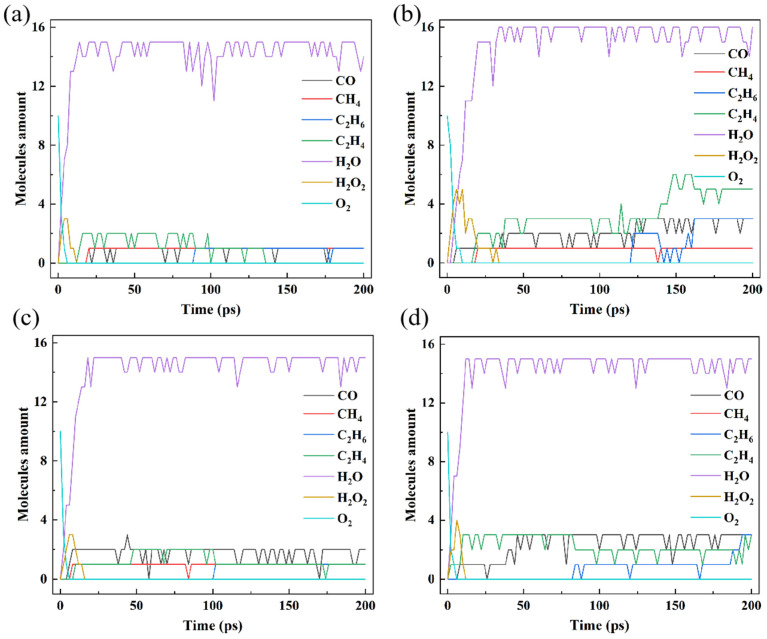
Changes in the amount of small molecules in (**a**) HDPE-C, (**b**) HDPE-A, (**c**) LDPE, (**d**) LLDPE.

Figure 3 shows the amount of small molecules in these PEs. Compared with the results of HDPE-C, HDPE-A produces more small molecules, such as CO and C_2_H_4_, indicating that PE with lower crystallinity is more susceptible to thermal oxidation. Comparing the results of HDPE-A, LDPE, and LLDPE, it is found that HDPE-A yields the most, LLDPE the second, and LDPE the lowest number of small molecules. It shows that under the same O_2_ concentration, unbranched and long-branched structures are more susceptible to thermal oxidation.

It is possible to directly uncover the mechanism of PE thermal oxidation by investigating small molecules that emerge or accumulate throughout the oxidation process. Figure 3 illustrates how H_2_O_2_ is produced in all systems by rapidly consuming O_2_. Subsequently, the H_2_O_2_ is consumed, suggesting that H_2_O_2_ is an essential intermediary product that may be related to the oxidation mechanism. Although H_2_O_2_ was not included in previous modeling work [15,16,18], it is present in photo-oxidation [38]. Our research demonstrates that H_2_O_2_ is an intermediate product that can be tough to identify experimentally because it exists for a short period of time. H_2_O is produced in substantial quantities when H_2_O_2_ is consumed, which makes sense since the final products of hydrocarbon oxidation are CO_2_ and H_2_O.

Moreover, C_2_H_4_, C_2_H_6_, CH_4_, and CO were generated but H_2_ and CO_2_ were not. This is because the initial quantity of O_2_ in this study is set small relative to the amount of C atoms to emphasize the differences in the interactions between various branched structures and O_2_. We analyzed the system of C_20_H_42_ + 32O_2_ to demonstrate the precision of the employed methodology, displaying the results in Figure S2. When there is enough O_2_ present, CO_2_ will be produced. Inadequate simulation time might be an explanation for the absence of H_2_ generation, as even in the experiment, little H_2_ gets generated [18].

**Figure 4:**
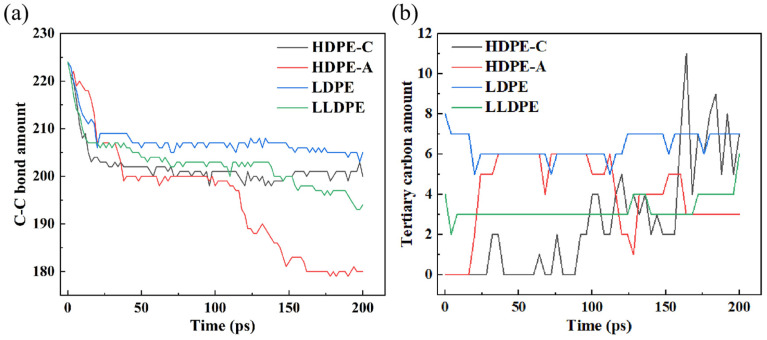
Changes of (**a**) C-C bond amount and (**b**) tertiary carbon amount.

It is reasonable that the degree of PE degradation may be inferred from the alteration in the quantity of C–C bonds [39]. Figure 4a illustrates that the C–C bonds in HDPE-A did not decrease much before 25 ps but decreased sharply after 100 ps, while the amount of C–C bonds in HDPE-C increased slowly after 100 ps. Figure 4b shows the change of tertiary carbon amount in four models. The number of tertiary carbon atoms in HDPE-C shows a tendency to increase gradually from the absence of tertiary carbon atoms, while in HDPE-A, tertiary carbon atoms are produced first and then decrease. The number of tertiary carbon atoms does not vary much in LDPE and LLDPE. Combined with the results of Figure 2a,b, the carbon chains in HDPE-C decreased at the late stage of thermal oxidation, while the number of carbon chains in HDPE-A gradually increased. It is hypothesized that the cross-linking of carbon chains occurs in HDPE-C, whereas the breakage of carbon chains in HDPE-A is more significant [12]. Accordingly, a decrease in crystallinity leads to a more complete carbon chain cleavage of PE, while a higher crystallinity facilitates carbon chain cross-linking. Compared with LDPE and LLDPE, the C–C bonds in HDPE-A are reduced the most, and the C–C bonds in LDPE are broken the least. This result is consistent with Figure 2 and Figure 3, indicating that the unbranched structure is more likely to be thermally oxidized, and the short and random branched chain structure is more stable.

### 3.2. Reaction Mechanism of Thermal Oxidation of PE

To reveal the reasons why crystallinity and branched chain structure lead to differences in thermal oxidation products of various PEs, this section first gives the thermal oxidation mechanism of PE through simulation at low temperature, pointing out that H_2_O_2_ is an important intermediate product. The details and thermodynamic energy barriers of the reactions of different PEs with O_2_ were then compared.

**Figure 5:**
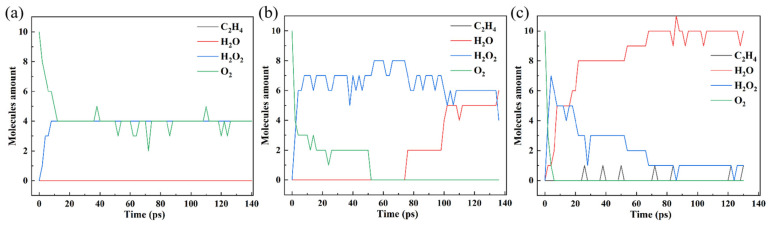
Changes in the amount of small molecules in HDPE-C system at (**a**) 1200 K, (**b**) 1600 K, (**c**) 2000 K.

Given that PE’s thermal oxidation process slows down at lower temperatures and that temperature merely changes the reaction rate rather than the reaction path, further research is needed to completely understand the PE thermal oxidation mechanism. As a result, we calculated the 140 ps oxidation process for the HDPE-C system at a lower temperature. Figure 5 depicts small molecule development throughout the system. At 1200 K, O_2_ consumption slows down, with only H_2_O_2_ formed but not consumed. When the temperature is raised to 1600 K, H_2_O is formed by the consumption of H_2_O_2_. At 2000 K, H_2_O is produced more quickly, and C_2_H_4_ starts to appear in the system. The oxidation products’ carbon number distribution also reveals that within 140 ps, the carbon chain breaks around 2000 K, and the generation of the C2, C4, and C10 products begins at 2000 K (Figure S3). It is evident that O_2_ initially reacts with the H atom on the carbon chain, producing an intermediate H_2_O_2_ when the thermal oxidation of PE takes place. The carbon chain breaks when H_2_O_2_ is reduced to H_2_O.

To acquire a better understanding of the oxidation mechanism of PE, it is necessary to analyze the details of H_2_O_2_ formation and consumption. Table 1 summarizes the main reactions involving O_2_ and species that include O in HDPE-C, exhibiting the H_2_O_2_ generation and consumption paths. The reactions involving H_2_O_2_ in HDPE-A, LDPE, and LLDPE systems are listed in Tables S1–S3, respectively. In essence, the chemical pathways resemble those of HDPE-C. This article will refer to the reaction that generates H_2_O as a simple reaction and the reaction that combines an O atom with a carbon chain as a complex reaction for the purpose of simplicity of description.

In HDPE-C, five O_2_ molecules immediately interact with the H atoms on the two neighboring C atoms on the carbon chain to produce H_2_O_2_. Through diffusion, the remaining O_2_ first interacts with a H atom to generate ·OOH, which subsequently combines with H atoms in other locations to form H_2_O_2_. H_2_O_2_ then breaks down into two ·OH or a reactive O atom as an intermediate product.

Because H_2_O_2_ displays a small O-O bond energy [40] and requires breaking more bonds to yield reactive O atoms, more ·OH is produced. Afterwards, these decomposition products react with PE. H_2_O emerges when an abundance of ·OH reacts with H atoms on the carbon chain, as Table 2 illustrates. The involvement of ·OH in the generation of H_2_O in HDPE-A, LDPE, and LLDPE is presented in Tables S4–S6. This process results in the formation of alkyl radicals or unsaturated C atoms. Figure S4 highlights the way certain ·OH and reactive O atoms interact with pre-existing alkyl radicals, leading to alcohols, which then transform into enols or CO. In HDPE-C, the transformation of enols into acids or ketones was not detected due to restricted calculation time. The details of the complex reactions in HDPE-A, LDPE, and LLDPE are shown in Figures S5–S10, respectively.

The mechanism of PE oxidation can be summarized as follows: O_2_ combines with H atoms on the carbon chain to form H_2_O_2_, and H_2_O_2_ decomposes to ·OH to continue dehydrogenating the carbon chain. Alkyl radicals are formed when the carbon chain is dehydrogenated, and these radicals eventually transform into more stable structures. During the reaction process, alkyl radicals may undergo H radical transfer reactions between carbon chains, or they may combine with O_2_, reactive O atoms, and ·OH to form more complex structures. The process of carbon chain evolution is complex and may be affected by numerous factors, such as the distribution of O-containing species, the free volume within the carbon chain, and the density distribution of the carbon chain.

There are two main views on the initiation reaction of PE thermal oxidation. Wu Chen et al. [41] and Lihua Chen et al. [16] used the ReaxFF calculation to show that PE thermal oxidation starts with C-H bond breakage, producing H radicals and carbon chain defects, which then react with O_2_ to form complex structures. Mingxiao Zhu et al. [17] employed the same method but found that it begins with C-C bond breakage, with the resulting defects reacting with O_2_. Both views agree that O_2_ does not participate in the reaction that forms defects but reacts with defects. However, our AIMD simulation suggests that O_2_ is involved in defect formation. To determine the correct view, we need to examine the energy barriers of C-H and C-C bond breaking and O_2_’s reaction with H atoms in PE using QC calculations. Xiangze Meng et al. [36] calculated the energy barrier for C-H bond breaking to be 388 kJ/mol. We will discuss the energy barriers for the other two reactions next.

**Figure 6:**
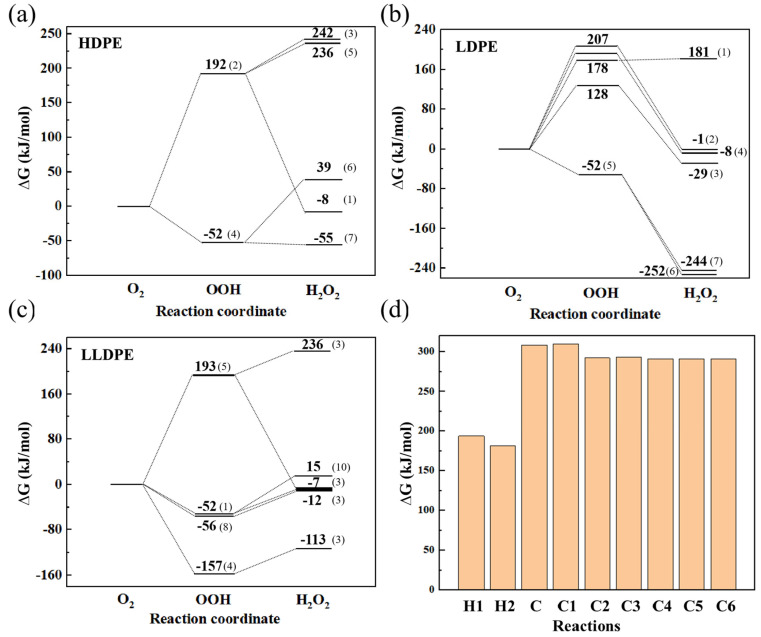
ΔG for H_2_O_2_ generation reaction in (**a**) HDPE-C, (**b**) LDPE, (**c**) LLDPE, for possible reactions (**d**) in PE systems. H1 represents the reaction of a H atom on unbranched PE with O_2_, H2 is the reaction of a H atom on a tertiary carbon in branched PE with O_2_. C represents a C-C bond break in unbranched PE, and Cx represents the breakage of a branched chain with x carbon atoms from the main chain.

As can be seen from the above tables, there are many PE fragments with different structures in each system that react with O_2_ to form H_2_O_2_. To avoid selective calculation of the reaction between a certain structure and O_2_, we performed QC calculations on all reactions that form H_2_O_2_ in Table 1 and Tables S2 and S3 and obtained the ΔG of these reactions to examine the influence of branched chains on the energy landscape of the H_2_O_2_ formation reaction. Note that since neither HDPE-C nor HDPE-A contains branched chains and the types of H_2_O_2_ formation reactions in these two systems are similar, only reactions in the HDPE-C system were selected to represent the unbranched structure. Since PE fragments of different structures are in the same system, there are various energy paths, as shown in Figure 6. Although they are different, these paths are processes that have actually occurred, and all paths need to be considered.

Since there is no transition state in C-C bond breakage, and the ΔG for the reaction of H atoms with O_2_ is close to the energy barrier evaluated from the transition state [36], we employed the ΔG to study the reaction’s difficulty.

The results in Figure 6 indicate that the process that produces ·OOH from O_2_ is the rate-determining step, even when certain reactions result in a reduction in the total energy landscape. The maximum ΔG of HDPE-C is 192 kJ/mol, LDPE 207 kJ/mol, and LLDPE 193 kJ/mol for the rate-determining step, similar to the results in Ref [36]. To study the thermodynamic landscape of PE thermal oxidation, we used QC calculations to obtain the ΔG for possible reactions. In Figure 6d, H1 represents the reaction of a H atom on unbranched PE with O_2_, H2 represents the reaction of a H atom on a tertiary carbon in branched PE with O_2_, C represents a C-C bond break in unbranched PE, and Cx represents the breakage of a branched chain with x carbon atoms from the main chain. Specific reaction details are presented in Figure S11. The results show that O_2_ reacts more readily with H atoms on PE chains than with carbon chain breaks. Specifically, H atoms on tertiary carbon react more readily (ΔG = 181.4 kJ/mol) with O_2_ compared to secondary carbon (ΔG = 193.8 kJ/mol), although the energy barrier is only 12.4 kJ/mol lower. Carbon chain breakage is much more difficult, with ΔG around 290–310 kJ/mol. These results indicate that O_2_ reacting with H atoms to form chemical defects has a lower energy barrier than C-H or C-C bond breaking. This contradicts previous results evaluated from the ReaxFF method, suggesting that AIMD is more accurate and reliable. Additionally, high-temperature simulations align with the PE thermal oxidation mechanism, as elevated temperatures do not trigger higher energy barrier processes prematurely, making high-temperature simulation a valid approach.

Figure S12 shows the ΔG for the reaction of H atoms on individual C atoms in branched chains of different lengths with O_2_. The results indicate that H atoms on primary carbons have the greatest difficulty reacting, followed by secondary and tertiary carbons. The presence of branched chains introduces both inert primary carbons and reactive tertiary carbons. Therefore, thermodynamically, O_2_ will more easily react with PE chains as the number of branched chains and tertiary carbons increases.

### 3.3. Effects of Branched Chains on PE Oxidation

This section starts with the analysis of the free volume between PE carbon chains and explores the distribution and diffusion behavior of O_2_ in four PEs. We also calculated the distribution of chemical defects and the carbon chain density distribution in all systems.

**Figure 7:**
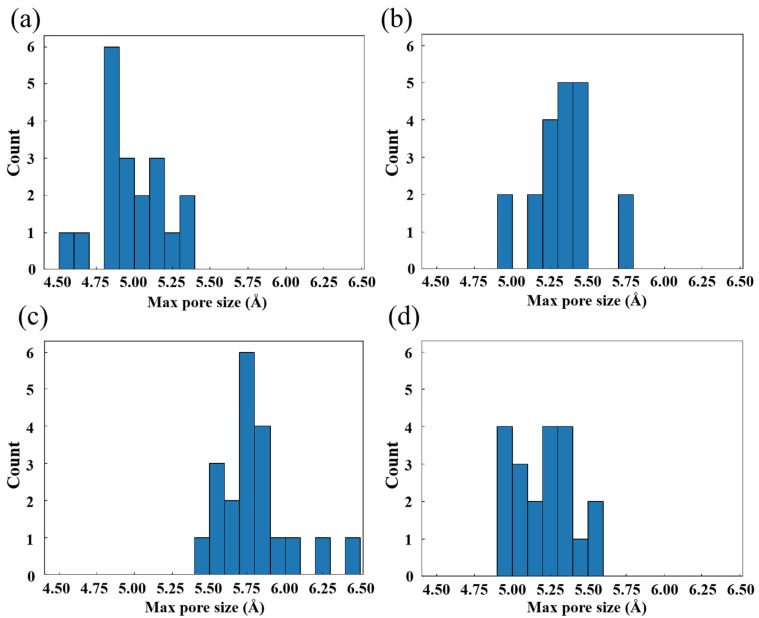
Maximum spherical pore size distribution in (**a**) HDPE-C, (**b**) HDPE-A, (**c**) LDPE, (**d**) LLDPE.

To characterize the continuous but irregular free volume between carbon chains, a sphere sufficiently large was searched to fit between the chains without contact with any H or C atoms. In the calculation, a frame of data is selected for every 12.5 fs, and the initial 250 fs time range is used to evaluate the initial maximum pore size of all systems. The results are presented in Figure 7. Among all systems, HDPE-C has the smallest maximum pore size of any system, approximately 4.8~5 Å. The maximum diameter of pores in HDPE-A is around 5.3~5.5 Å, indicating that higher crystallinity is not conducive to the formation of large free volume. LDPE has the greatest maximal pore size, ranging from 5.75 to 5.9 Å. This might be because LDPE has random and short branches, allowing larger free volume to emerge due to intermolecular repulsion between various chains, while the maximum diameter in LLDPE is approximately 5.2~5.4 Å.

We analyzed the free volume size and shape of three amorphous PEs. Free volumes in HDPE-C are not discussed as they are clearly regular orifices. Results for HDPE-A, LDPE, and LLDPE are shown in Figures S13–S15. Most free volumes in amorphous PE are below 5 Å^3^. LDPE has the highest free volume percentage at 4.4%, while HDPE-A and LLDPE are around 4%. HDPE-A has the largest free volume at 138.7 Å^3^, characterized by connected, narrow spaces. LDPE’s largest free volume is 108.3 Å^3^, with homogeneous macropores. LLDPE’s largest free volume is 54.7 Å^3^, with both homogeneous and narrow spaces. The shape affects the distribution and diffusion of O_2_ in PE.

**Figure 8:**
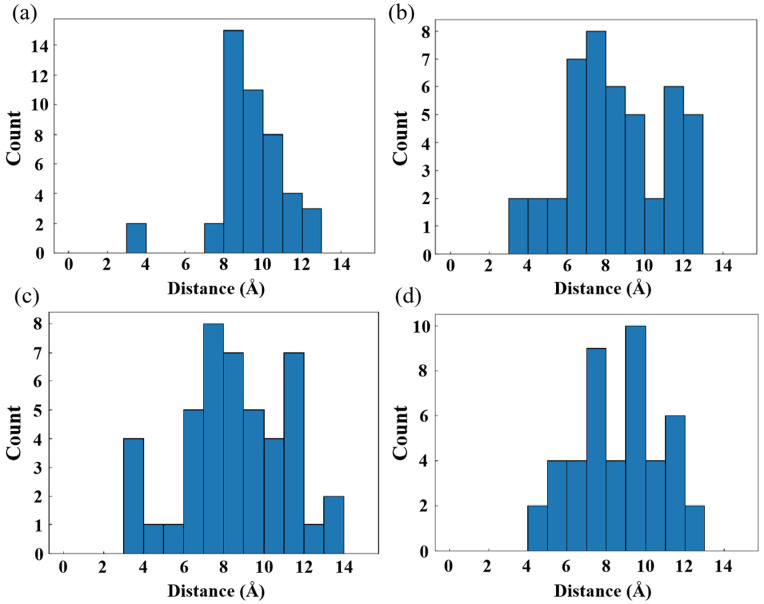
(**a**) HDPE-C, (**b**) HDPE-A, (**c**) LDPE, (**d**) LLDPE oxygen spacing distribution diagram at the initial moment.

The entropy effect of O_2_ within the free volume varies depending on the size of the free volumes. Figure 8 displays the distribution diagram of the distance between all O_2_ molecules in each system at the beginning of the simulation. A system with a uniform distribution of O_2_ will have a single primary peak that is positioned in the middle of the picture, similar to a normal distribution curve. Uneven O_2_ distribution is indicated by the presence of multiple peaks. The distribution of O_2_ is uneven in HDPE-A but uniform in HDPE-C, indicating that a disordered structure leads to disordered O_2_ distribution. Among all disordered systems, the O_2_ distribution in LDPE and HDPE-A with larger free volume is uneven, showing that the larger free volume will lead to an irregular O_2_ distribution.

**Figure 9:**
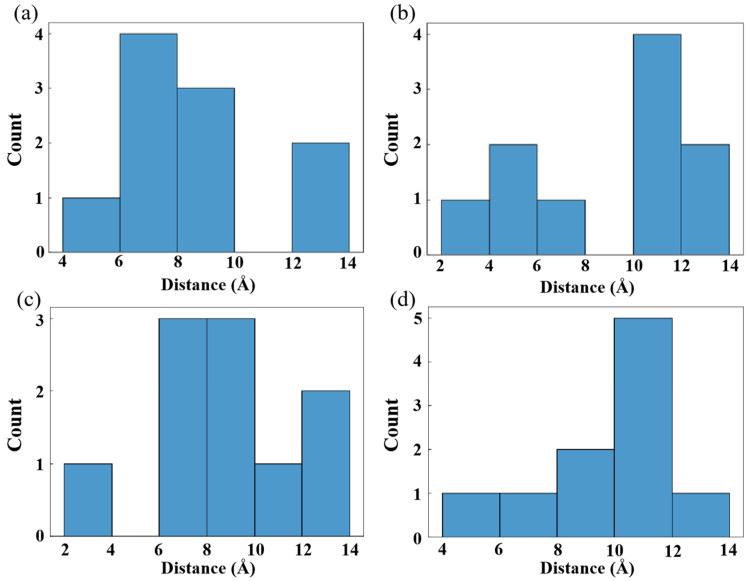
O_2_ diffusion displacement distribution in (**a**) HDPE-C, (**b**) HDPE-A, (**c**) LDPE, (**d**) LLDPE.

Even though the carbon chain structure will result in variations in the homogeneity of the O_2_ distribution in each framework, O_2_ will diffuse before reacting with PE. This influences the location of the PE-O_2_ reaction, and the carbon chain’s structure additionally regulates the diffusion process. The distance between each O_2_ molecule in each system from the location at the beginning time to the position at the reaction time was evaluated, which was referred to as the diffusion displacement to characterize the diffusion route length of O_2_ prior to interacting with PE. Figure 9 illustrates the distribution of O_2_ diffusion displacement throughout all systems. The average diffusion displacement of O_2_ in HDPE-C and HDPE-A systems is 8.61 Å and 9.05 Å, respectively. This is because HDPE-C has regular channel structures, and as O_2_ molecules prefer to migrate in the direction of the channels, there is relatively little diffusion displacement [36]. The average O_2_ diffusion displacement in LDPE and LLDPE systems is 8.72 Å and 9.85 Å, respectively. This difference is thought to be caused by greater free volume in LDPE, which contributes to an uneven distribution of carbon atom density (Figure S16). Regions with higher carbon atom density are not conducive to the diffusion of O_2_.

To investigate the effect of free volume on O_2_ diffusion in amorphous PE, we calculated the distance-displacement ratio (DDR) of O_2_ diffusion. DDR reflects the diffusion pattern by comparing the total distance O_2_ traverses before reacting to its displacement from the initial position to the reaction site. Figure S17 shows that DDR is smallest in HDPE-A, largest in LDPE, and intermediate in LLDPE. This indicates that in HDPE-A, O_2_ moves directly to reaction sites due to narrow free volumes, leading to dispersed defects. In LDPE, O_2_ undergoes significant meandering motion in homogeneous macropores, leading to concentrated defects. MSD results (Figure S18) support this, showing norm diffusion for O_2_ in HDPE-A and LLDPE, while LDPE exhibits subdiffusion-like behavior.

The distribution of reaction sites between O_2_ and PE directly affects the distribution of chemical defects on the PE carbon chain. Since chemical defects play a promoter role in the subsequent carbon chain breaking reaction, understanding the distribution of chemical defects is of great significance for analyzing the breakage of PE carbon chains.

**Figure 10:**
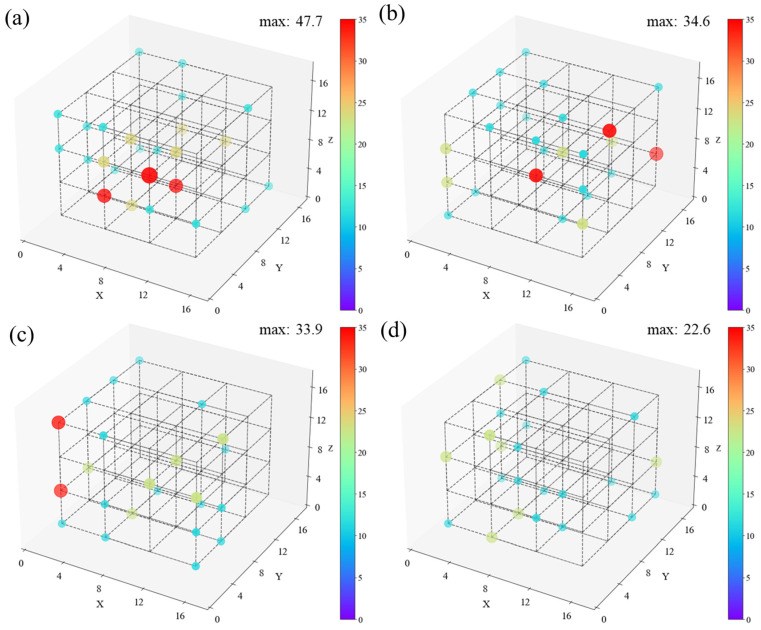
Distribution of chemical defects in (**a**) HDPE-C, (**b**) HDPE-A, (**c**) LDPE, (**d**) LLDPE system at t = 50 ps (unit: nm^−3^). From red to purple, the chemical defect density decreases from high to low.

This work attempts to give the spatial distribution of chemical defects at 50 ps. The rationale behind adopting this moment is depicted in Figure 4. The initial stage of the C-C bond breaking and the PE-O_2_ reaction terminate at around 50 ps. Defects in the carbon chain are the primary triggers of subsequent cleavage, which predominantly originate from chemical defects. Note that each system was split into 4 × 4 × 4 small parts to determine the density distribution of chemical defects, as shown in Figure 10.

The atomic density of each region was calculated and represented by the size and color of the point at the region’s center. Points are not shown in regions with zero density. Neighboring points are connected with dotted lines to illustrate their relative positions. Figure 10 shows that the chemical defect distribution in HDPE-C and HDPE-A is relatively uniform, indicating crystallinity does not affect it. In contrast, LDPE shows an uneven distribution of chemical defects. HDPE-A and LLDPE show no spatial preference for the distribution of chemical defects.

**Figure 11:**
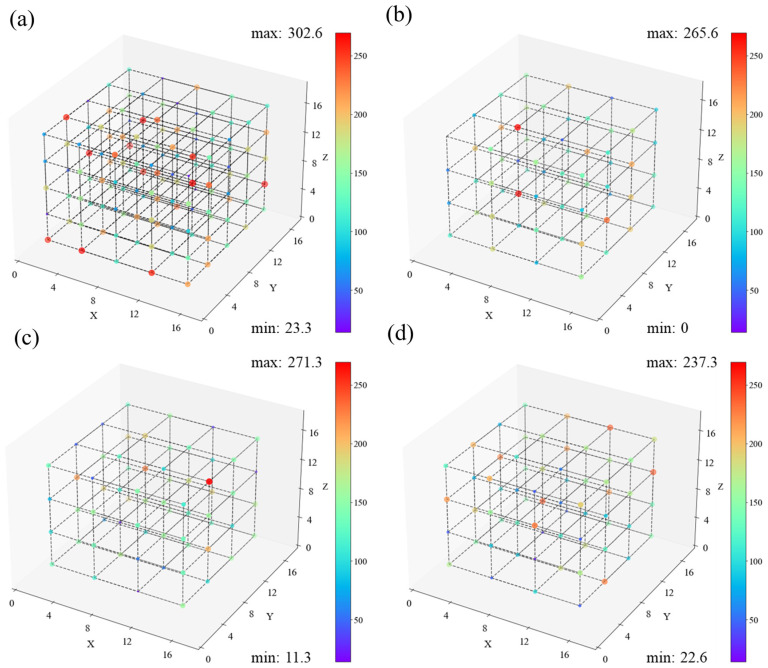
Distribution of atoms in (**a**) HDPE-C, (**b**) HDPE-A, (**c**) LDPE, (**d**) LLDPE system at t = 50 ps (unit: nm^−3^). From red to purple, the density decreases from high to low.

To analyze how the density distribution of chemical defects affects PE chain cracking, we need the density distribution of C and H atoms in the PE chain. Figure 11 shows the spatial distribution of C and H atom densities at 50 ps. Each point’s color and size represent the atomic density of a sub-region. LDPE has a location with an exceptionally high atomic density of 271.3 nm^−3^. Other locations range from 100 nm^−3^ or less, with about five places reaching 200 nm^−3^. The carbon chain atom density distribution for other systems is essentially homogeneous and overlaps with the chemical defects distribution.

According to the description above, the impact of the branched-chain structure on the cracking behavior of PE is random, and short branched chains lead to the formation of large pores between PE chains, causing O_2_ to distribute around the pores and react with carbon chains locally, resulting in the free radical density distribution staggered with the carbon chain atomic density distribution, and delaying atomic migration and carbon chain cleavage.

**Figure 12:**
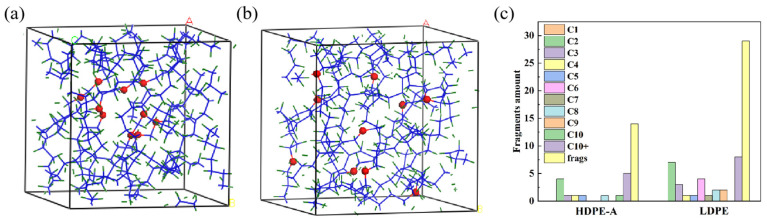
Artificially designed (**a**) concentrated defect distribution for HDPE-A, (**b**) scattered defect distribution for LDPE (all defects are shown in red, C atoms in blue, H atoms in green), and (**c**) corresponding product distribution at the end of 100 ps of 2400 K molecular dynamics simulation.

To verify the above conclusions and demonstrate the impact of chemical defect distribution on PE bond breaking, we created ten concentrated defects in the HDPE-A model and ten dispersed defects in the LDPE model by removing H atoms from the carbon chain in Figure 12. After removing all O_2_ molecules, we conducted a 100 ps dynamics simulation at 2400 K. Figure 12c shows the product distribution: LDPE degrades more significantly, producing the most C1 and C6 products, while HDPE-A produces the most C1 and C10+ products, indicating only preliminary bond breaking. LDPE’s total fragment amount is twice that of HDPE-A, suggesting that dispersed defects lead to more severe carbon chain breaks.

The greater degradation of HDPE-A compared to LDPE, as seen in Figure 2 and Figure 3, is due to more thorough O_2_ diffusion in HDPE-A and concentrated movement within cavities in LDPE. This results in a uniform distribution of chemical defects in HDPE-A and a concentrated distribution in LDPE. When defects are artificially concentrated in HDPE-A and dispersed in LDPE, LDPE degrades more significantly than HDPE-A, thus justifying our conjecture that a more dispersed distribution of chemical defects leads to more drastic degradation. It is believed that the PE carbon chain breakage can be delayed by adjusting the polymerization process and processing parameters to gain higher crystallinity and an ideal branched chain structure to achieve a localized distribution of defects.

To explain why LDPE is generally more unstable than HDPE-A under thermo-oxidative conditions, we conducted dynamics calculations on relaxed models using the NVT ensemble and investigated the interaction energies between PE and O_2_, presented in Table S7. The interaction energy per molecule for HDPE-A is 3.687 kJ/mol, while for LDPE, it is 2.242 kJ/mol. These results differ from published work [36] due to the higher O_2_ density used in our simulations (0.0959 g/cm^3^ vs. atmospheric 0.0016 g/cm^3^). Despite this, the results show that compared with HDPE-A, LDPE can accommodate O_2_ more readily. If the dissolved equilibrium O_2_ concentration in HDPE-A as a reference is taken into consideration, LDPE’s concentration would be 1.79 times higher, contributing to its greater instability.

## 4. Conclusions

The SCC-DFTB method is utilized in this work to simulate the thermal oxidation of crystallized and amorphous PE with various branched chain structures. The thermal oxidation products of these PEs are compared. After assessing the ΔG of the reaction between various PEs and O_2_ and analyzing the distribution of chemical defects and carbon chain density, the following conclusions were achieved that show crystallized PE is more prone to cross-linking reactions instead of chain scission during thermal oxidation.

Compared with HDPE-A, the number of carbon chains in HDPE-C decreased during the thermal oxidation, while the number of C-C bonds increased, and the number of tertiary carbon atoms at the end of the reaction was twice that of HDPE-A, indicating that cross-linking reactions occurred. This phenomenon may be due to its higher crystallinity. Among the three amorphous systems, the carbon chain breakage of HDPE-A is more complete, followed by LLDPE and LDPE. In order to explain this strange phenomenon, we first analyzed the details of the reactions occurring in each system and found that the process of PE’s thermal oxidation begins with the combination of O_2_ and H atoms on the carbon chain, with H_2_O_2_ as the intermediate product. In these systems, the energy barrier that needs to be overcome to generate H_2_O_2_ is approximately 190 kJ/mol, and the difference caused by branching is not significant. The decomposition of H_2_O_2_ produces hydroxyl radicals, which then react with the carbon chain to produce H_2_O or alcohol structures.

By examining these specific reactions that have occurred and those that may occur through QC calculations, it was found that the branched chain introduces reactive tertiary H atoms (with energy barrier of 180 kJ/mol when reacting with O_2_) and inert primary H atoms (with the energy barrier of 210 kJ/mol when reacting with O_2_), but does not have much effect on the energy landscape of the reaction of the O_2_ with PEs. By analyzing the molecular structures of various PEs, we found that branching introduces large pores with a size of about 6 Å, leading to an uneven distribution of O_2_. This also restricts the diffusion of O_2_, concentrating the reaction sites between O_2_ and PE, forming localized chemical defects. This concentration delays the subsequent breakage of PE chains. In contrast, the free volume in the unbranched PE system exhibits elongated and uneven pore structures, providing pathways for the diffusion of O_2_, which results in a more uniform distribution of reaction sites between O_2_ and PE. Chemical defects, such as the reaction sites for the subsequent chain scission in PE, will lead to faster and more thorough carbon chain breakage when distributed uniformly.

## Figures and Tables

**Table 1:** Reactions involving O_2_ and the formation and consumption of H_2_O_2_ in HDPE-C.

Index	Reaction	Amount
1	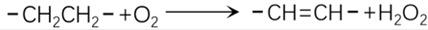	5
2	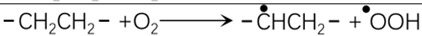	2
3	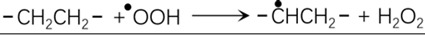	2
4	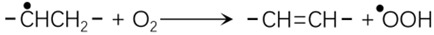	2
5	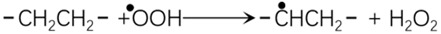	1
6	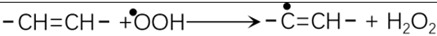	1
7	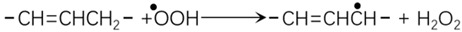	1
8	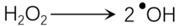	7
9	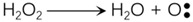	2

**Table 2:** Simple reactions involving ·OH radicals in HDPE-C.

Index	Reaction	Amount
1	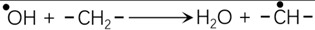	6
2	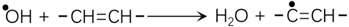	2
3	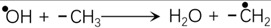	1
4	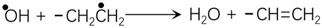	1
5	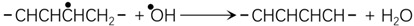	1
6	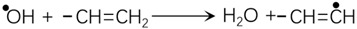	1

## Data Availability

Data will be made available on request.

## Supplementary Materials

The following supporting information can be downloaded at: https://www.mdpi.com/article/10.3390/polym16213038/s1, Figure S1: Carbon number distribution of various polyethylene oxidation products at the end of simulation; Figure S2: (a) Carbon number distribution of oxidation products and (b) variation of the amount of small molecules in the C_20_H_42_ + 32O_2_ system; Figure S3: Evolution of carbon number distribution of thermal oxidation products of HDPE-C system at (a) 1200K, (b) 1600K, (c) 2000K; Figure S4: Complex reactions involving O_2_ in HDPE-C; Figure S5: Complex reactions involving O_2_ in HDPE-A; Figure S6: Complex reactions involving ·OH in HDPE-A; Figure S7: Complex reactions involving O_2_ in LDPE; Figure S8: Complex reactions involving ·OH in LDPE; Figure S9: Complex reactions involving O_2_ in LLDPE; Figure S10: Complex reactions involving ·OH in LLDPE; Figure S11: The reactions mentioned in QC calculations. The index x-y shows the position of H atom that reacts with O_2_; Figure S12: The Gibbs free energy change for O_2_ reacting with branched chain’s H atom. For index x-y, x means the C atoms amount of branched chain, and y means the C atomic number that provides H atoms on the branched chain. The larger y represents the further C atom from the main chain; Figure S13: Free volume of HDPE-A. The (a) free volume and corresponding amount distribution, (b) shape of largest free volume, (c) shape of 2^nd^-largest free volume, (d) shape of 3^rd^-largest free volume; Figure S14: Free volume of LDPE. The (a) free volume and corresponding amount distribution, (b) shape of largest free volume, (c) shape of 2^nd^-largest free volume, (d) shape of 3^rd^-largest free volume; Figure S15: Free volume of LLDPE. The (a) free volume and corresponding amount distribution, (b) shape of largest free volume, (c) shape of 2^nd^-largest free volume, (d) shape of 3^rd^-largest free volume; Figure S16: C and H atom density distribution at the initial moment in (a) HDPE-C, (b) HDPE-A, (c) LDPE, (d) LLDPE system (unit: nm^−3^); Figure S17: The diffusion distance displacement ratio for O_2_ in (a) HDPE-A, (b) LDPE, (c) LLDPE system; Figure S18: Mean square displacement (MSD) calculation for amorphous PE systems; Table S1: Generation and consumption of H_2_O_2_ in HDPE-A; Table S2: Formation and consumption of H_2_O_2_ in LDPE; Table S3: Formation and consumption of H_2_O_2_ in LLDPE; Table S4: Simple reactions involving ·OH in HDPE-A; Table S5: Simple reactions involving ·OH in LDPE; Table S6: Simple reactions involving ·OH in LLDPE; Table S7: The interaction energy (*E_inter_*) of PE and O_2_ and dissolution O_2_ concentration comparison for HDPE-A and LDPE system.
