# Supplementary material for: Effects of Crystallinity and Branched Chain on Thermal Degradation of Polyethylene: A SCC-DFTB Molecular Dynamics Study
Source: Polymers (Basel). 2024 Oct 29;16(21):3038. doi: 10.3390/polym16213038 (PMC11548203; doi:10.3390/polym16213038)
Supplement: Supplementary file 1 [file polymers-16-03038-s001.zip › polymers-3264448-supplementary.pdf]

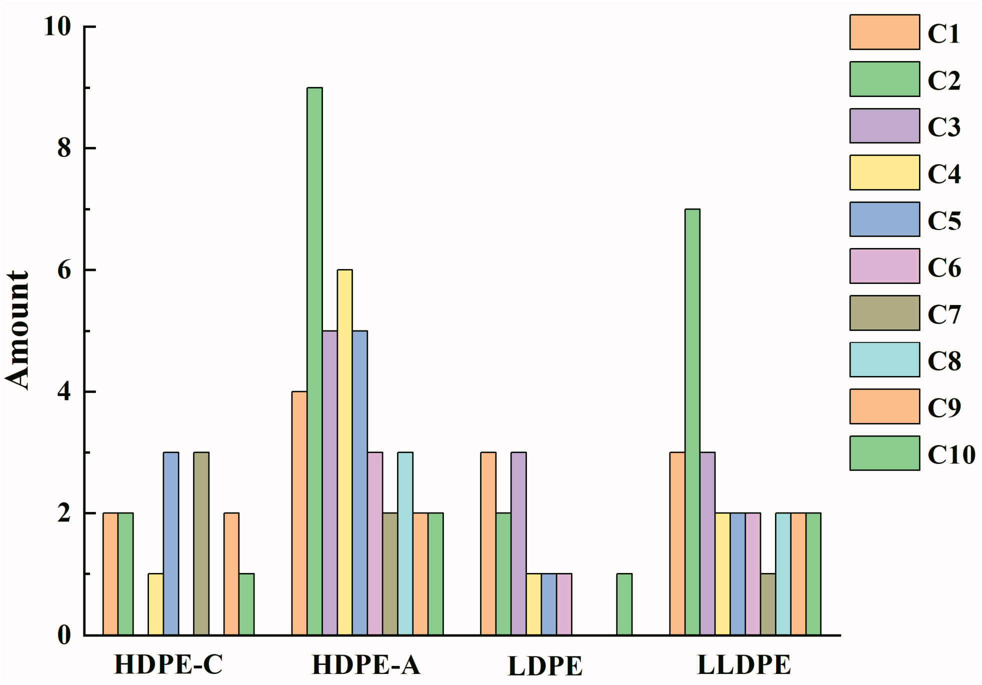

Figure S1. Carbon number distribution of various polyethylene oxidation products at the end of simulation.

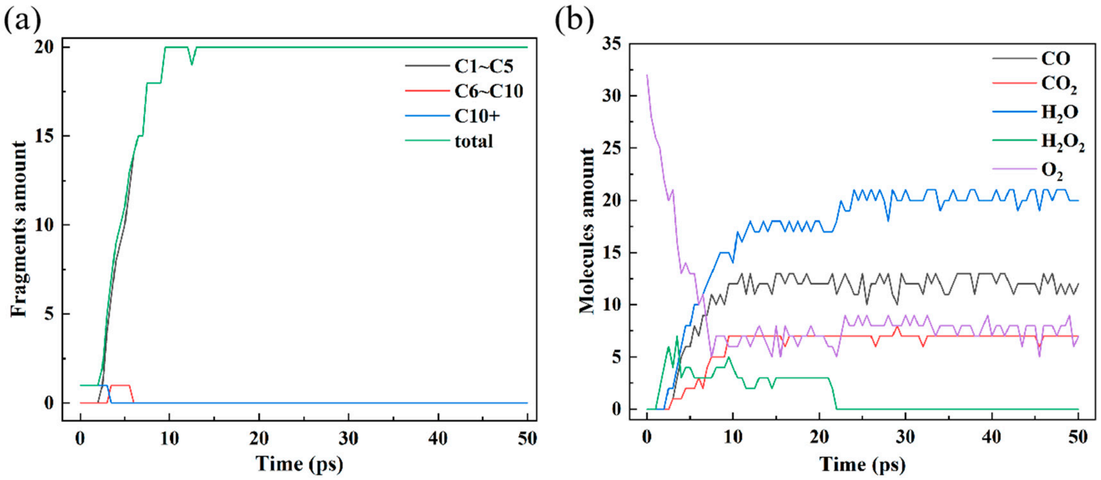

Figure S2. (a) Carbon number distribution of oxidation products and (b) variation of the amount of small molecules in the $C_{20}H_{42}$+$32O_2$ system.

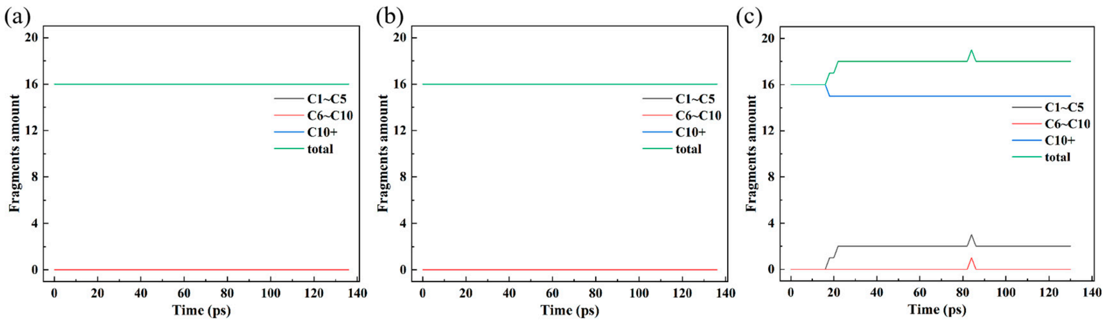

Figure S3. Evolution of carbon number distribution of thermal oxidation products of HDPE-C system at (a) 1200K, (b) 1600K, (c) 2000K.

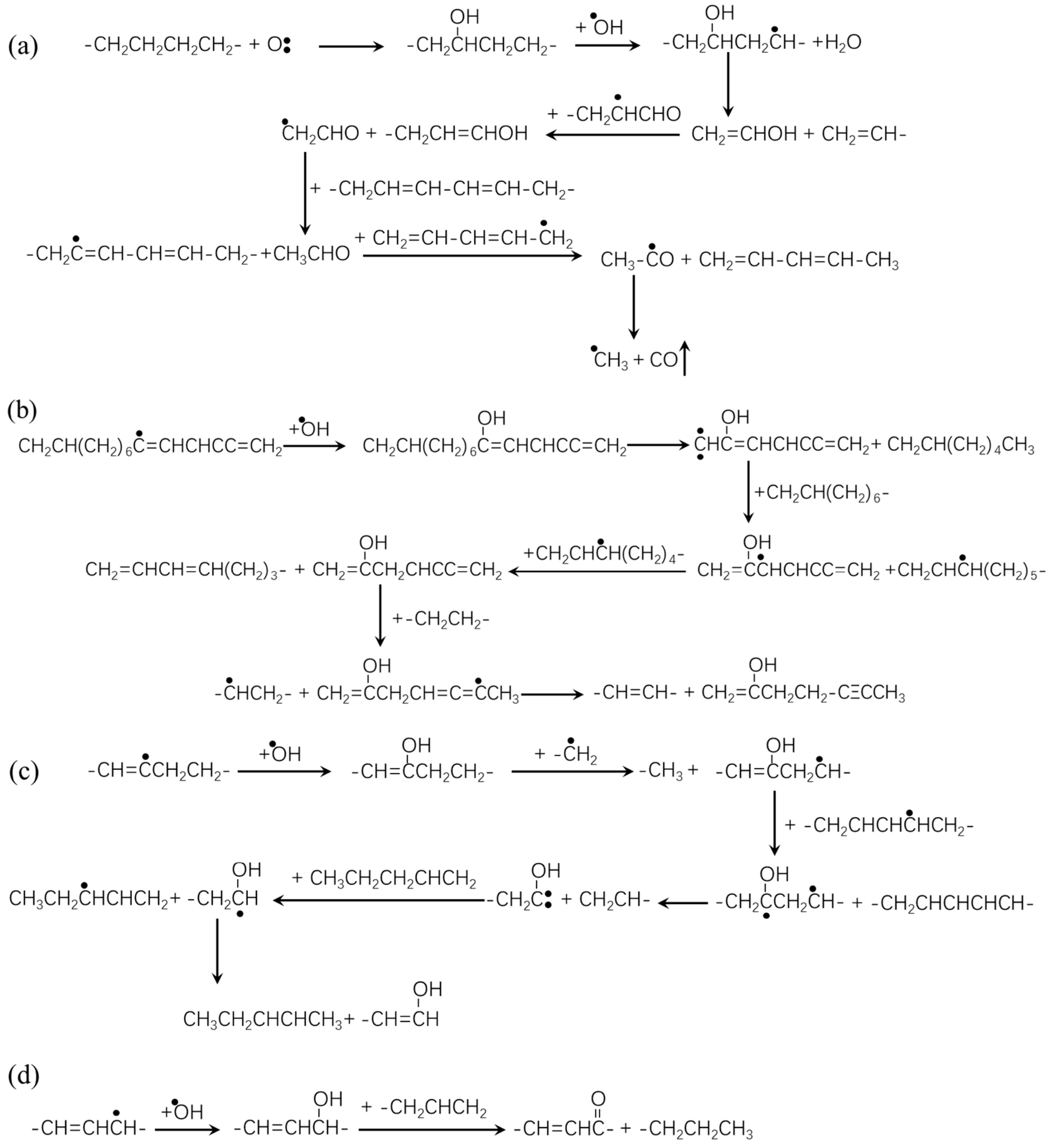

Figure S4. Complex reactions involving O_2 in HDPE-C.

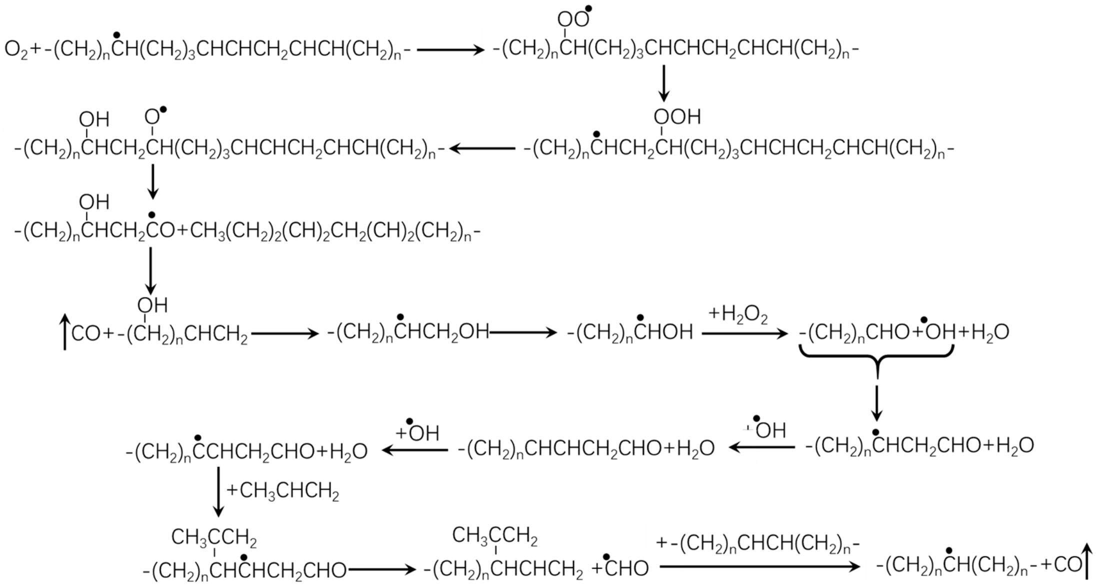

Figure S5. Complex reactions involving O_2 in HDPE-A.

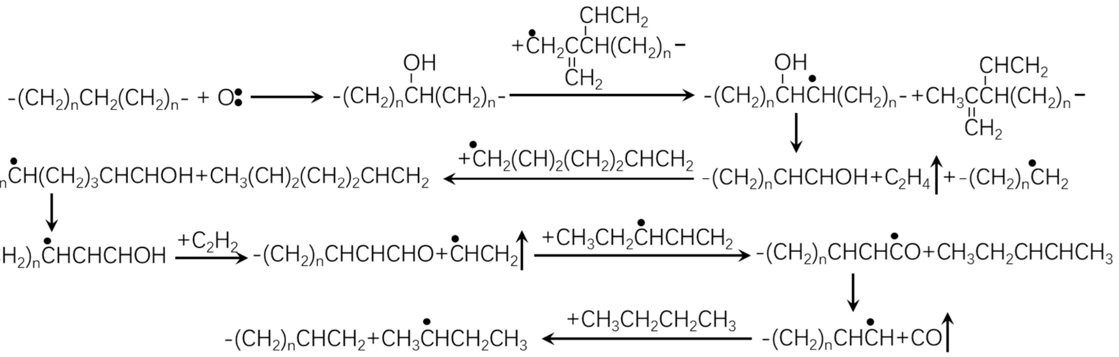

Figure S6. Complex reactions involving ·OH in HDPE-A.

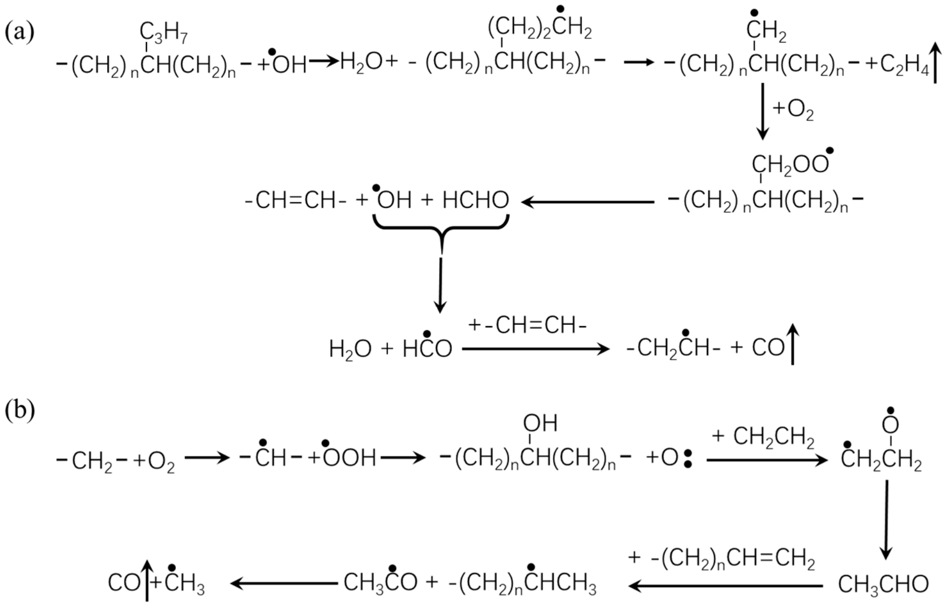

Figure S7. Complex reactions involving O_2 in LDPE.

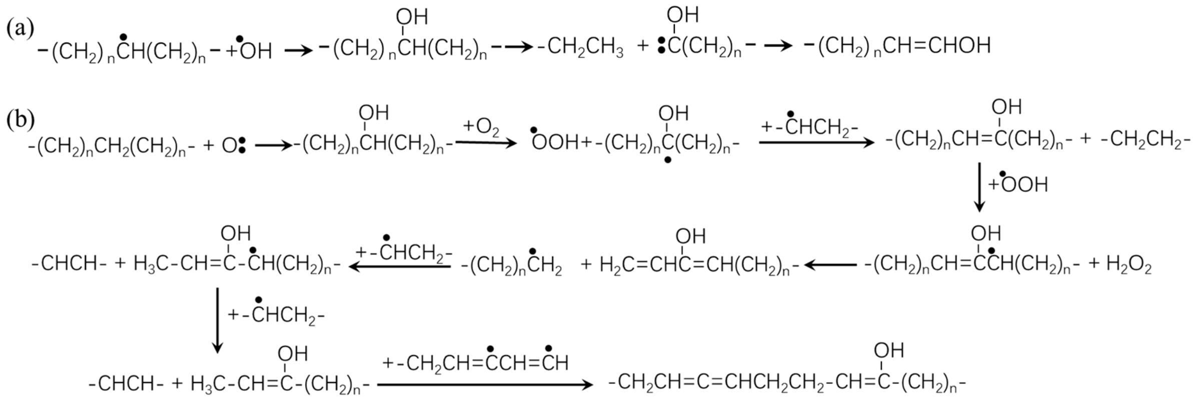

Figure S8. Complex reactions involving ·OH in LDPE.

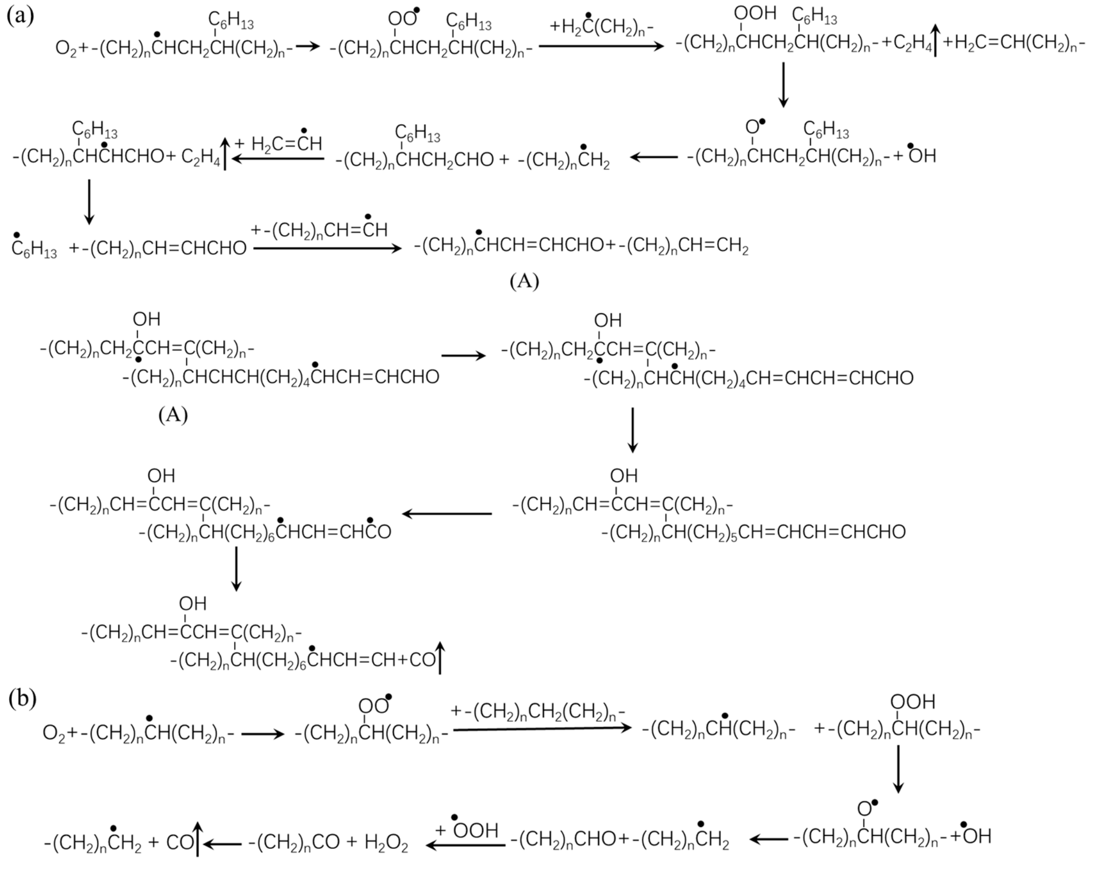

Figure S9. Complex reactions involving O_2 in LLDPE.

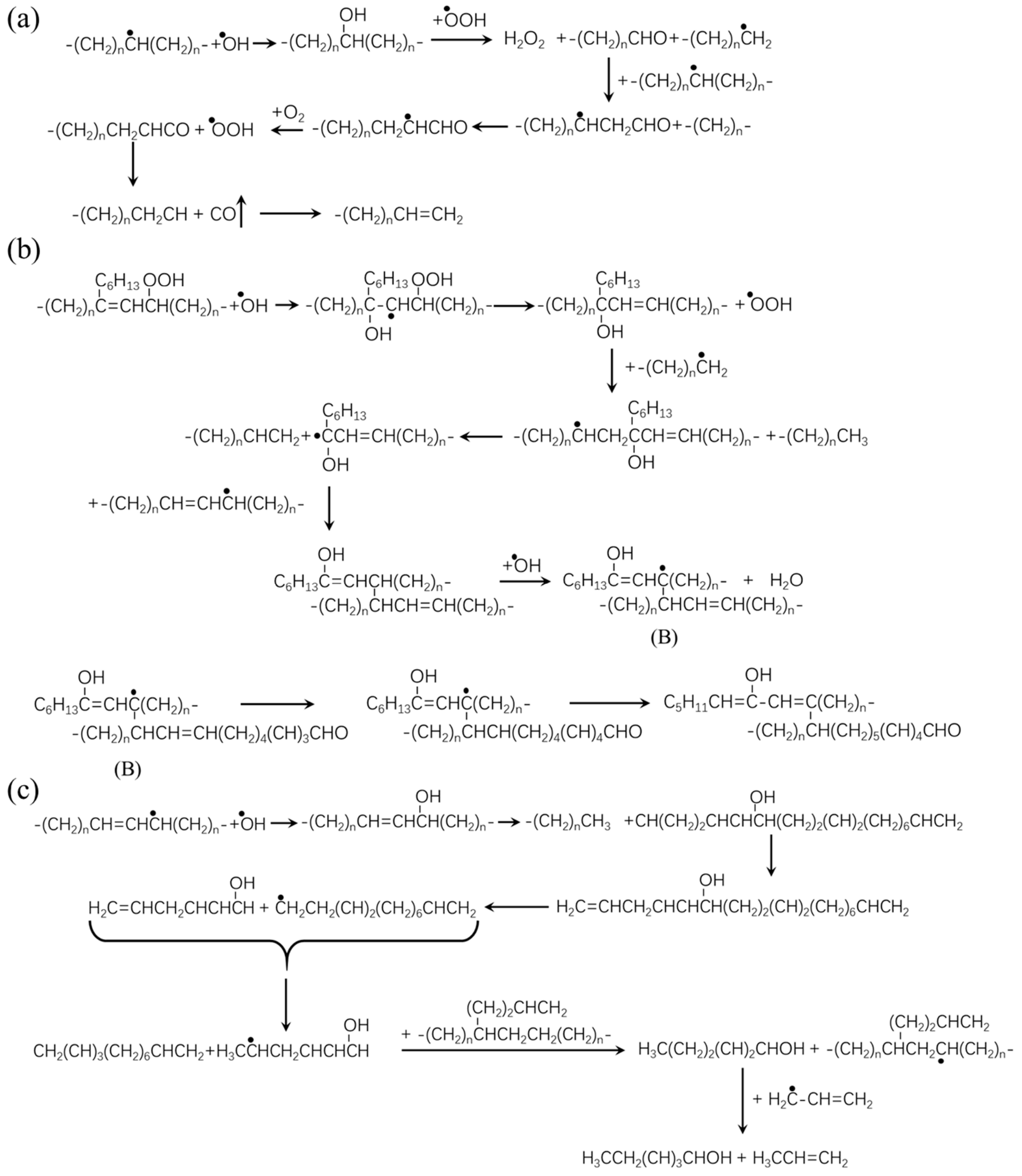

Figure S10. Complex reactions involving ·OH in LLDPE.

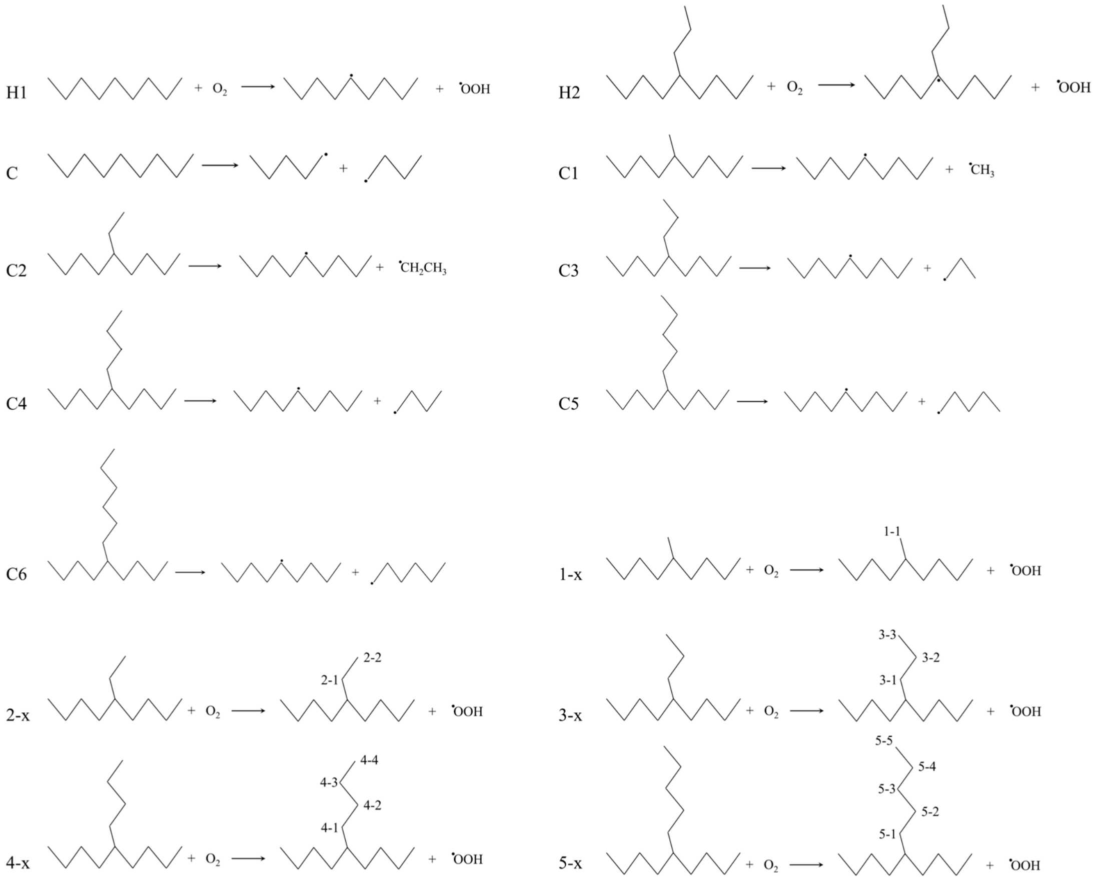

Figure S11. The reactions mentioned in QC calculations. The index x-y shows the position of H atom that reacts with O_2.

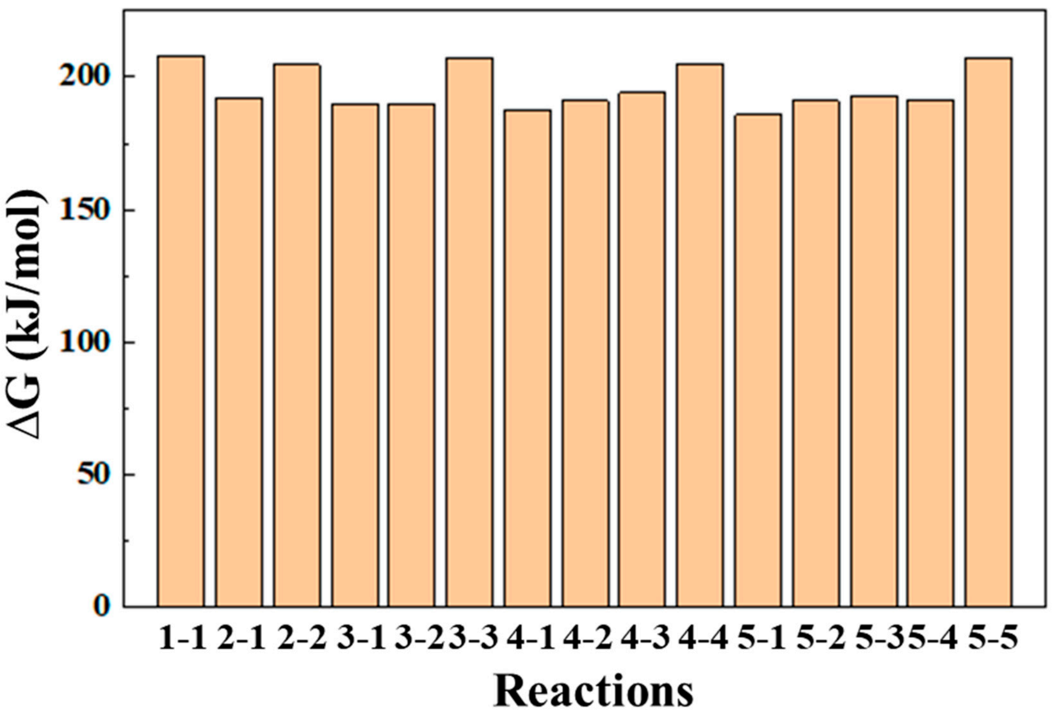

Figure S12. The Gibbs free energy change for O_2 reacting with branched chain's H atom. For index x-y, x means the C atoms amount of branched chain, and y means the C atomic number that provides H atoms on the branched chain. The larger y represents the further C atom from the main chain.

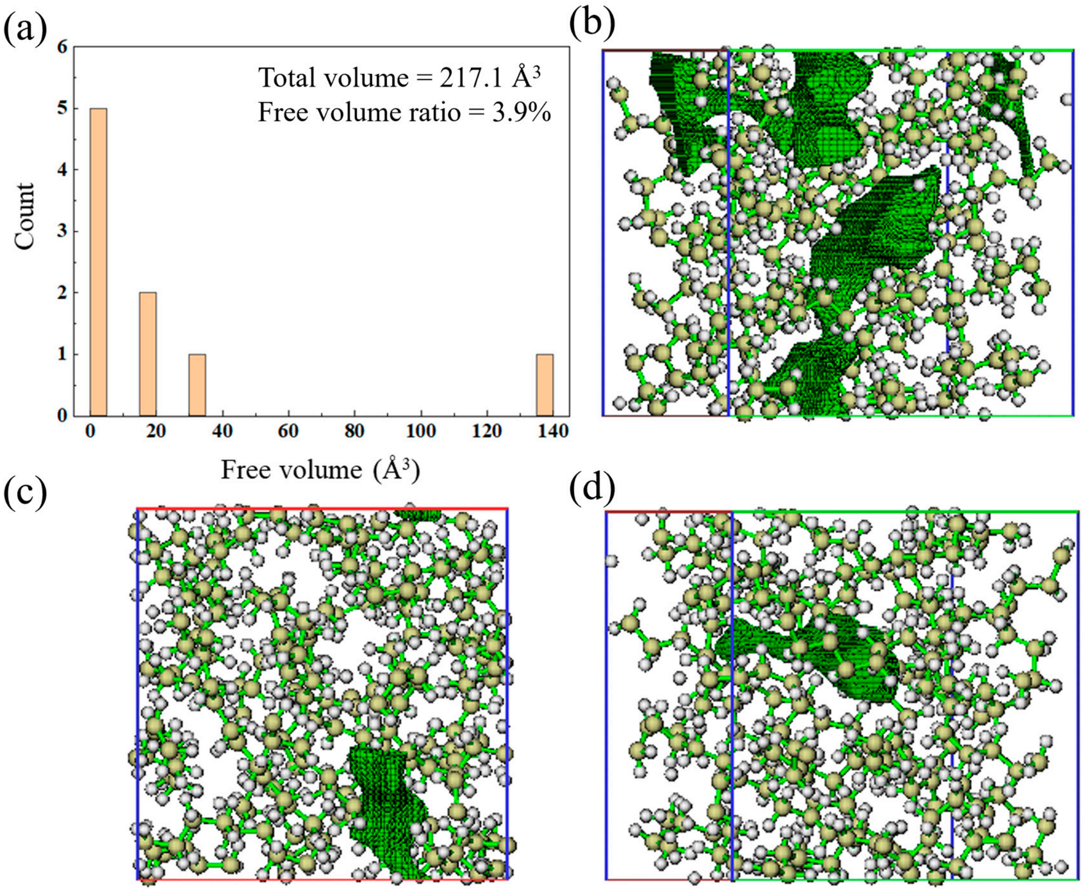

Figure S13. Free volume of HDPE-A. The (a) free volume and corresponding amount distribution, (b) shape of largest free volume, (c) shape of 2nd-largest free volume, (d) shape of 3rd-largest free volume.

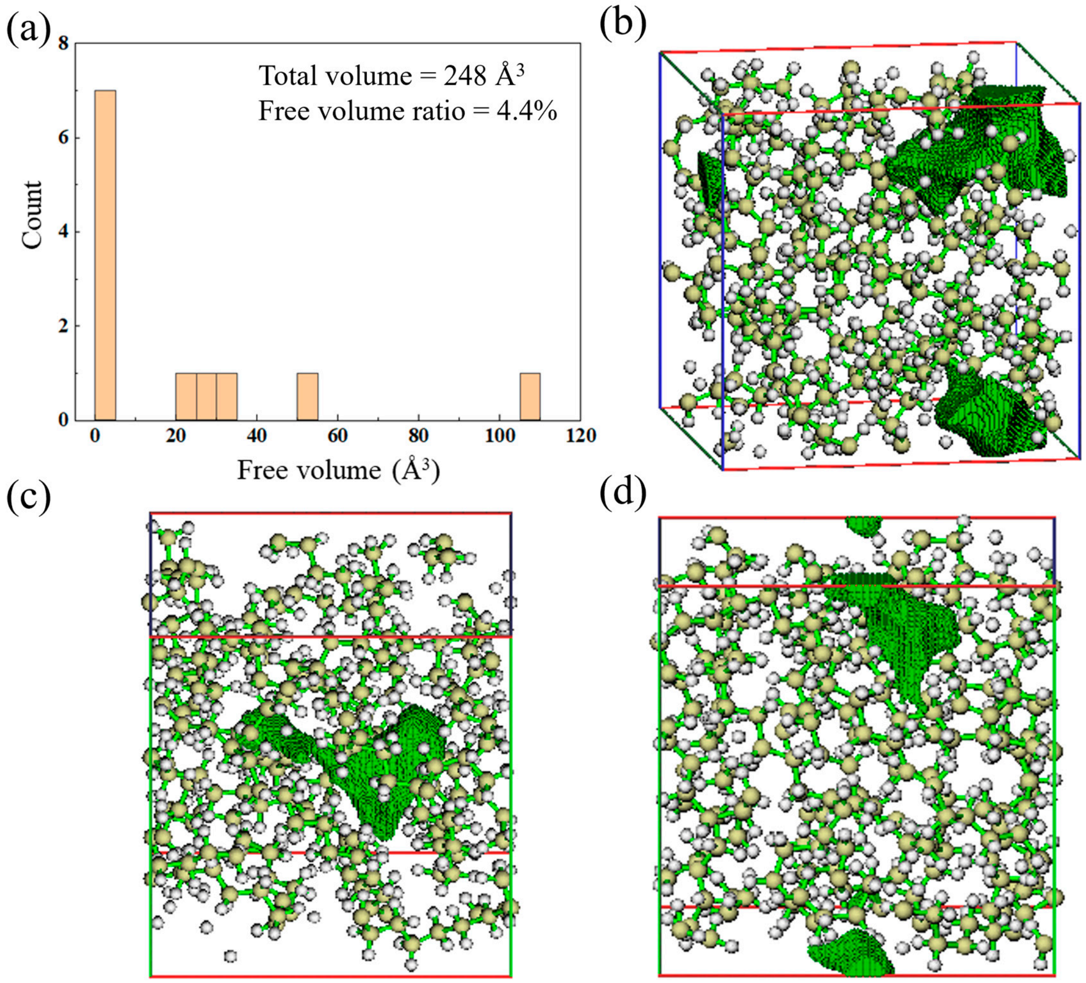

Figure S14. Free volume of LDPE. The (a) free volume and corresponding amount distribution, (b) shape of largest free volume, (c) shape of 2nd-largest free volume, (d) shape of 3rd-largest free volume.

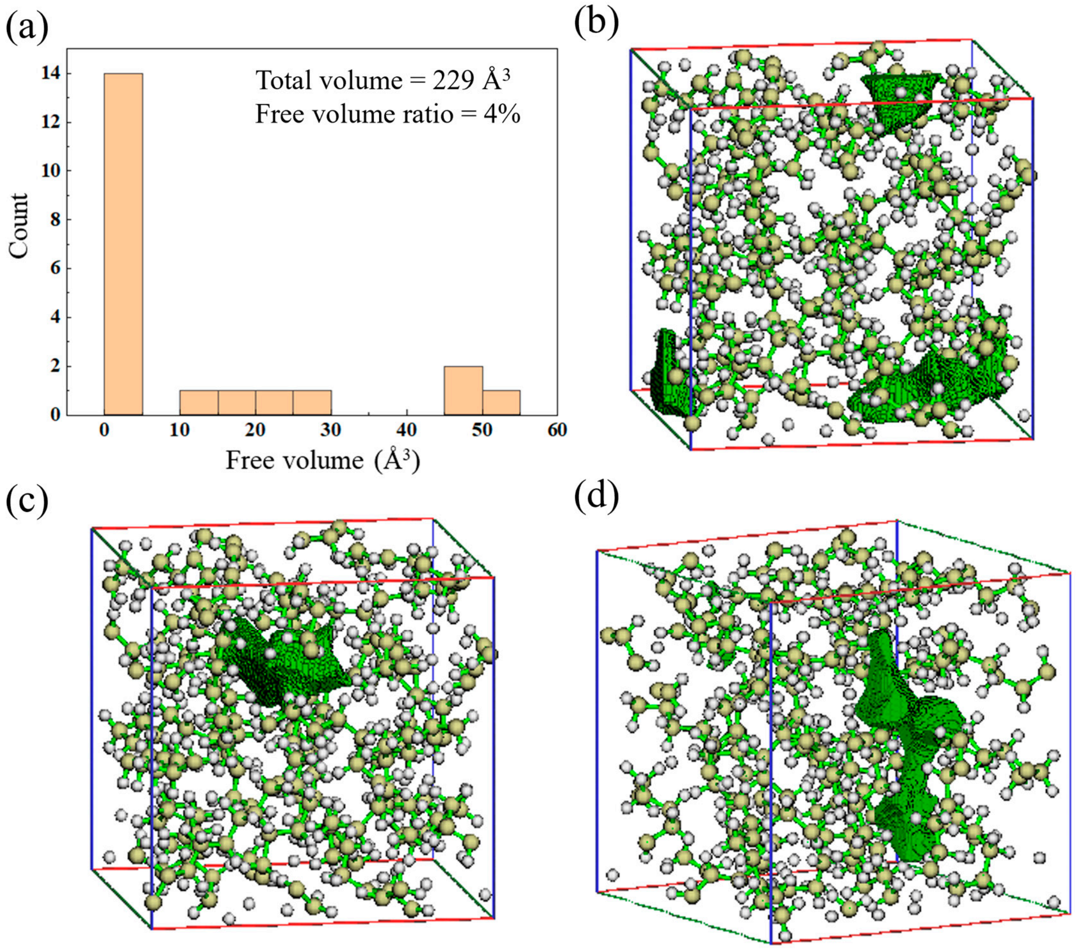

Figure S15. Free volume of LLDPE. The (a) free volume and corresponding amount distribution, (b) shape of largest free volume, (c) shape of 2nd-largest free volume, (d) shape of 3rd-largest free volume.

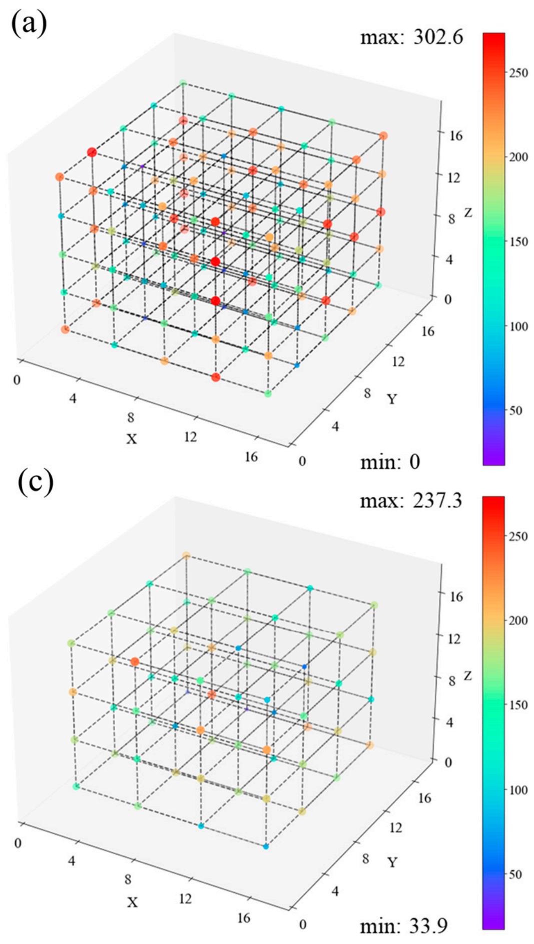

Figure S16. C and H atom density distribution at the initial moment in (a) HDPE-C, (b) HDPE-A, (c) LDPE, (d) LLDPE system (unit: nm^{-3}).

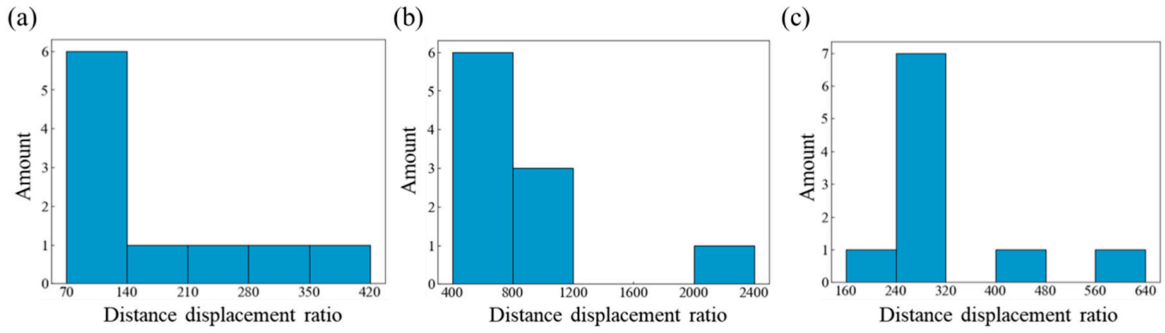

Figure S17. The diffusion distance displacement ratio for O_2 in (a)HDPE-A, (b)LDPE, (c)LLDPE system.

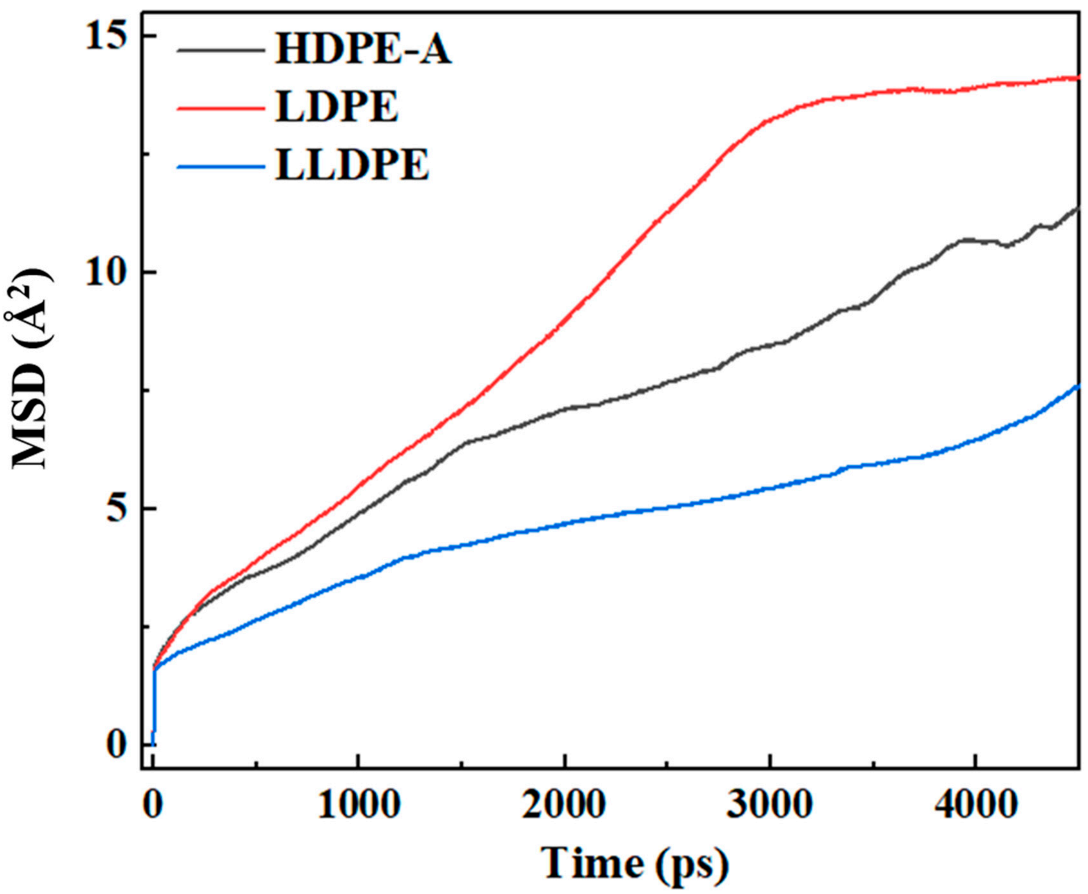

Figure S18. Mean square displacement (MSD) calculation for amorphous PE systems.

Table S1. Generation and consumption of H_2O_2 in HDPE-A.

Index	Reaction	Amount
1	$-\dot{C}HCH_2- + O_2 \longrightarrow -CH{=}CH- + {}^{\bullet}OOH$	4
2	$-(CH_2)_n- + O_2 \longrightarrow -(CH_2)_n\dot{C}H(CH_2)_n- + {}^{\bullet}OOH$	3
3	$-CH_2CH_2\dot{C}HCH_2- + O_2 \longrightarrow -CH{=}CH\dot{C}HCH_2- + H_2O_2$	1
4	$-CH_2CH_2- + O_2 \longrightarrow -CH{=}CH- + H_2O_2$	1
5	$-\dot{C}HCH_2- + O_2 \longrightarrow -CHCH- + {}^{\bullet}OOH$	1
6	$-(CH_2)_n- + {}^{\bullet}OOH \longrightarrow -(CH_2)_n\dot{C}H(CH_2)_n- + H_2O_2$	4
7	$-\dot{C}HCH_2- + {}^{\bullet}OOH \longrightarrow -CH{=}CH- + H_2O_2$	2
8	$2\,{}^{\bullet}OOH \longrightarrow O_2 + {}^{\bullet}OH$	1
9	$-(CH_2)_n\dot{C}HOH + H_2O_2 \longrightarrow -(CH_2)_nCHO + H_2O + {}^{\bullet}OH$	1
10	$H_2O_2 \longrightarrow 2\,{}^{\bullet}OH$	6
11	$H_2O_2 \longrightarrow H_2O + O\colon$	1

Table S2. Formation and consumption of H_2O_2 in LDPE.

Index	Reaction	Amount
1	$-(CH_2)_8\overset{OH}{C}H(CH_2)_3- + O_2 \longrightarrow -(CH_2)_8\overset{OH}{\underset{\bullet}{C}}(CH_2)_3- + {}^{\bullet}OOH$<br>$-(CH_2)_8\overset{OH}{\underset{\bullet}{C}}(CH_2)_3- + -CH_2\dot{C}HCH_2- \longrightarrow -(CH_2)_7CH{=}\overset{OH}{C}(CH_2)_3- + -(CH_2)_3-$<br>$-(CH_2)_7CH{=}\overset{OH}{C}(CH_2)_3- + {}^{\bullet}OOH \longrightarrow -(CH_2)_7CH{=}\overset{OH}{\underset{\bullet}{C}}CH(CH_2)_2- + H_2O_2$	1
2	$-(CH_2)_n\overset{C_3H_7}{C}H(CH_2)_n- + O_2 \rightarrow -(CH_2)_n\overset{(CH_2)_2\dot{C}H_2}{C}H(CH_2)_n- + {}^{\bullet}OOH \rightarrow -(CH_2)_n\overset{CH_2CH{=}CH_2}{C}H(CH_2)_n- + H_2O_2$	1
3	$-CH_2CH{=}CHCH_2CH_2\overset{C_2H_5}{C}H(CH_2)_n- + O_2 \rightarrow -CH_2CH{=}CHCH{=}CH\overset{C_2H_5}{C}H(CH_2)_n- + H_2O_2$	1
4	$-CH_2CH_2- + O_2 \longrightarrow -CH{=}CH- + H_2O_2$	2
5	$-CH_2\dot{C}H- + O_2 \longrightarrow -CH{=}CH- + {}^{\bullet}OOH$	2
6	$-CH_2\dot{C}H- + {}^{\bullet}OOH \longrightarrow -CH{=}CH- + H_2O_2$	1
7	$CH_3\dot{C}H(CH_2)_n- + {}^{\bullet}OOH \longrightarrow CH_2{=}CH(CH_2)_n- + H_2O_2$	1
8	$-CH_2- + {}^{\bullet}OOH \longrightarrow -\dot{C}H- + H_2O_2$	2
9	$O_2 + H_2O_2 \longrightarrow 2\,{}^{\bullet}OOH$	1
10	$H_2O_2 \longrightarrow 2\,{}^{\bullet}OH$	7
11	$H_2O_2 \longrightarrow H_2O + O\colon$	1

Table S3. Formation and consumption of H_2O_2 in LLDPE.

Index	Reaction	Amount
1	$O_2 + -CH_2\dot{C}H- \longrightarrow {}^{\bullet}OOH + -CH{=}CH-$	3
2	${}^{\bullet}OOH + -(CH_2)_nCH{=}CH(CH_2)_n- \longrightarrow H_2O_2 + -(CH_2)_nCH{=}CH(CH_2)_3\dot{C}H-$	1
3	${}^{\bullet}OOH + -(CH_2)_nCH_2(CH_2)_n- \longrightarrow H_2O_2 + -(CH_2)_n\dot{C}H(CH_2)_n-$	5
4	$O_2 + -(CH_2)_n\dot{C}HCHO \longrightarrow {}^{\bullet}OOH + -(CH_2)_nCH{=}CO$	1
5	$O_2 + -(CH_2)_nCH_2(CH_2)_n- \longrightarrow {}^{\bullet}OOH + -(CH_2)_n\dot{C}H(CH_2)_n-$	2
6	$O_2 + -CH_2CH_2CH_3 \longrightarrow {}^{\bullet}OOH + -CH_2\dot{C}HCH_3$	1
7	${}^{\bullet}OOH + -CH_2\dot{C}HCH_3 \longrightarrow H_2O_2 + -CH{=}CHCH_3$	1
8	$O_2 + -(CH_2)_n\overset{C_6H_{13}}{C}HCH_2\dot{C}H(CH_2)_n- \longrightarrow {}^{\bullet}OOH + -(CH_2)_n\overset{C_6H_{13}}{C}HCH{=}CH(CH_2)_n-$	1
9	$-(CH_2)_n\overset{OOH}{C}H\dot{C}H\overset{C_6H_{13}}{C}H(CH_2)_n- + {}^{\bullet}OOH \longrightarrow -(CH_2)_n\overset{OOH}{C}HCH{=}\overset{C_6H_{13}}{C}(CH_2)_n- + H_2O_2$	1
10	${}^{\bullet}OOH + -(CH_2)_n\overset{OH}{C}H(CH_2)_n- \longrightarrow H_2O_2 + -(CH_2)_nCHO + -(CH_2)_n\dot{C}H_2$	1
11	$H_2O_2 + {}^{\bullet}OH \longrightarrow H_2O + {}^{\bullet}OOH$	1
12	$H_2O_2 \longrightarrow 2\,{}^{\bullet}OH$	7
13	$H_2O_2 + -(CH_2)_nCH_2\dot{C}H_2 \longrightarrow H_2O + -(CH_2)_nCH{=}CH_2 + {}^{\bullet}OH$	1

Table S4. Simple reactions involving ·OH in HDPE-A.

Index	Reaction	Amount
1	$-(CH_2)_n- + {}^{\bullet}OH \longrightarrow -(CH_2)_n\dot{C}H(CH_2)_n + H_2O$	3
2	$-(CH_2)_n\dot{C}H(CH_2)_n- + {}^{\bullet}OH \longrightarrow -(CH_2)_nCHCH(CH_2)_n + H_2O$	3
3	$-(CH_2)_3CHCH_2 + {}^{\bullet}OH \longrightarrow -(CH_2)_3CH\dot{C}H + H_2O$	1
4	$-(CH_2)_nCH_2CHCHCHCH_2 + {}^{\bullet}OH \longrightarrow -(CH_2)_n\dot{C}HCHCHCHCH_2 + H_2O$	1
5	$-(CH_2)_n(CH)_2CH_2(CH)_2(CH_2)_2CH_3 \xrightarrow{+{}^{\bullet}OH} -(CH_2)_n(CH)_2\dot{C}H(CH)_2(CH_2)_2CH_3 + H_2O$	1
6	$-(CH_2)_nCHCH(CH_2)_n- + {}^{\bullet}OH \longrightarrow -(CH_2)_n\dot{C}CH(CH_2)_n + H_2O$	1
7	$-(CH_2)_n\dot{C}HCH_2- + {}^{\bullet}OH \longrightarrow -(CH_2)_nCHCH- + H_2O$	1
8	$-(CH_2)_nCH_2CH_2CHO + {}^{\bullet}OH \longrightarrow -(CH_2)_n\dot{C}HCH_2CHO + H_2O$	1
9	$-(CH_2)_n\dot{C}HCH_2CHO + {}^{\bullet}OH \longrightarrow -(CH_2)_nCHCHCH_2CHO + H_2O$	1
10	$-(CH_2)_nCHCHCH_2CHO + {}^{\bullet}OH \longrightarrow -(CH_2)_n\dot{C}CHCH_2CHO + H_2O$	1

Table S5. Simple reactions involving ·OH in LDPE.

Index	Reaction	Amount
1	$-CH_2- + \dot{O}H \longrightarrow -\dot{C}H- + H_2O$	4
2	$-(CH_2)_n\overset{C_3H_7}{C}H(CH_2)_n- + \dot{O}H \longrightarrow -(CH_2)_n\overset{CH_2\dot{C}HCH_3}{C}H(CH_2)_n- + H_2O$	2
3	$-(CH_2)_7CH_2CH{=}CH_2 + \dot{O}H \longrightarrow -(CH_2)_7\dot{C}HCH{=}CH_2 + H_2O$	1
4	$-(CH_2)_6\dot{C}HCH_3 + \dot{O}H \longrightarrow -(CH_2)_6CH{=}CH_2 + H_2O$	1
5	$-CH_2\overset{CH=CH_2}{C}HCH_2CH{=}CH_2 + \dot{O}H \longrightarrow -\dot{C}H\overset{CH=CH_2}{C}HCH_2CH{=}CH_2 + H_2O$	1
6	$CH_3(CH_2)_3CH{=}CH_2 + \dot{O}H \longrightarrow CH_3(CH_2)_3\dot{C}{=}CH_2 + H_2O$	1
7	$-(CH_2)_n\dot{C}HCH_3 + \dot{O}H \longrightarrow -(CH_2)_nCH{=}CH_2 + H_2O$	1
8	$-(CH_2)_nCH{=}CH\dot{C}H(CH_2)_n- + \dot{O}H \longrightarrow -(CH_2)_nCH{=}CH\underset{\bullet}{\dot{C}}(CH_2)_n- + H_2O$	1
9	$HCHO + \dot{O}H \rightarrow \dot{C}HO + H_2O\uparrow \xrightarrow{+\,-CH=CH-} CO\uparrow + -CH_2\dot{C}H-$	1
10	$-(CH_2)_n\overset{C_3H_7}{C}H(CH_2)_n- + \dot{O}H \longrightarrow -(CH_2)_n\overset{CH_2CH_2\dot{C}H_2}{C}H(CH_2)_n- + H_2O$	1

Table S6. Simple reactions involving ·OH in LLDPE.

Index	Reaction	Amount
1	$-(CH_2)_nCH_2(CH_2)_n- + \dot{O}H \longrightarrow -(CH_2)_n\dot{C}H(CH_2)_n- + H_2O$	3
2	$CH_3CH_2CH{=}CH_2 + \dot{O}H \longrightarrow CH_3CH_2\dot{C}{=}CH_2 + H_2O$	1
3	$-CHCHCH_2CH_2- + \dot{O}H \longrightarrow -CHCHCH_2\dot{C}H- + H_2O$	1
4	$-(CH_2)_nCHCH_2 + \dot{O}H \longrightarrow -(CH_2)_nCH\dot{C}H + H_2O$	1
5	$-(CH_2)_nCH_2CHCH_2 + \dot{O}H \longrightarrow -(CH_2)_n\dot{C}HCHCH_2 + H_2O$	1
6	$-(CH_2)_n\dot{C}HCH_2(CH_2)_n- + \dot{O}H \longrightarrow -(CH_2)_nCH{=}CH(CH_2)_n- + H_2O$	1
7	$-(CH_2)_n\overset{C_6H_{13}}{C}HCH_2CH_2(CH_2)_n- + \dot{O}H \longrightarrow -(CH_2)_n\overset{C_6H_{13}}{C}HCH_2\dot{C}H(CH_2)_n- + H_2O$	1
8	$-(CH_2)_n\overset{OH}{C}{=}CH\overset{(CH_2)_n}{C}H\underset{(CH_2)_n}{C}HCH{=}CH(CH_2)_n- + \dot{O}H \longrightarrow -(CH_2)_n\overset{OH}{C}{=}CH\underset{\bullet}{\overset{(CH_2)_n}{C}}\underset{(CH_2)_n}{C}HCH{=}CH(CH_2)_n- + H_2O$	1
9	$-(CH_2)_nCH_2CHCH\underset{OH}{\overset{C_6H_{13}}{C}}(CH_2)_n- + \dot{O}H \longrightarrow -(CH_2)_n\dot{C}HCHCH\underset{OH}{\overset{C_6H_{13}}{C}}(CH_2)_n- + H_2O$	1

Table S7. The interaction energy (E_{inter}) of PE and O_2 and dissolution O_2 concentration comparison for HDPE-A and LDPE system.

System	E_{inter} (kJ/mol)	C_{O_2}(relative)
HDPE-A	3.687	1
LDPE	2.242	1.79
